# A Platform for Spatiotemporal “Matrix” Stimulation in Brain Networks Reveals Novel Forms of Circuit Plasticity

**DOI:** 10.3389/fncir.2021.792228

**Published:** 2022-01-05

**Authors:** Nathan R. Wilson, Forea L. Wang, Naiyan Chen, Sherry X. Yan, Amy L. Daitch, Bo Shi, Samvaran Sharma, Mriganka Sur

**Affiliations:** ^1^Department of Brain and Cognitive Sciences, Massachusetts Institute of Technology, Cambridge, MA, United States; ^2^Nara Logics, Inc., Boston, MA, United States; ^3^Program in Computational and Systems Biology, Massachusetts Institute of Technology, Cambridge, MA, United States; ^4^Department of Electrical Engineering and Computer Science, Massachusetts Institute of Technology, Cambridge, MA, United States

**Keywords:** sequence, cortical, plasticity, network, calcium imaging, multi-electrode, stimulation, spatiotemporal

## Abstract

Here we demonstrate a facile method by which to deliver complex spatiotemporal stimulation to neural networks in fast patterns, to trigger interesting forms of circuit-level plasticity in cortical areas. We present a complete platform by which patterns of electricity can be arbitrarily defined and distributed across a brain circuit, either simultaneously, asynchronously, or in complex patterns that can be easily designed and orchestrated with precise timing. Interfacing with acute slices of mouse cortex, we show that our system can be used to activate neurons at many locations and drive synaptic transmission in distributed patterns, and that this elicits new forms of plasticity that may not be observable via traditional methods, including interesting measurements of associational and sequence plasticity. Finally, we introduce an automated “network assay” for imaging activation and plasticity across a circuit. Spatiotemporal stimulation opens the door for high-throughput explorations of plasticity at the circuit level, and may provide a basis for new types of adaptive neural prosthetics.

## Introduction

While brain networks are optimized for pattern detection, and organized by patterned activity (Sur and Rubenstein, 2005), methods for defining and delivering parallel stimulation patterns to multiple loci in neuronal networks using controlled experimental parameters have remained largely under-developed. Meanwhile, and especially in the past decade, methods for recording activity in neuronal circuits have expanded from “one neuron at a time” via patch clamp and single unit recording to sophisticated population readouts capable of monitoring the pattern of activity across multiple neurons simultaneously, such as multi-electrode recording (Chapin et al., 1999; Donoghue, 2002; Segev et al., 2004; Gollisch and Meister, 2008; Nicolelis, 2008; Lubenov and Siapas, 2009) and calcium imaging (Garaschuk et al., 2000; Stosiek et al., 2003; Ohki et al., 2005; Schummers et al., 2008). In contrast, systems for stimulating many sites in a brain circuit in orchestrated spatiotemporal patterns have, for the most part, remained locked in the “one locus at a time” paradigm. To continue this shift toward network physiology at the circuit level, we sought a straightforward “population stimulation” platform—a system to not only measure populations of neurons, but to further engage and activate them in multi-site ensembles via causal experimental regimes.

A growing number of neurological processes are believed to arise not only from the activation of single neurons or loci, but from the concerted interplay of neurons across a network (Pouget et al., 2000). Such “population” activation of multiple adjacent loci has been shown to underpin such diverse phenomena as sensation (Laurent and Davidowitz, 1994; Runyan et al., 2017), locomotion (Stent et al., 1978), spatial processing (Foster and Wilson, 2006), song production (Yu and Margoliash, 1996; Long and Fee, 2008), feature recognition (Pasupathy and Connor, 2002), and decision making (Carrillo-Reid et al., 2019; Daie et al., 2021). Moreover, concurrent multi-cellular dynamics form the basis of pivotal recent theories on general brain processing, such as cortical songs (Ikegaya et al., 2004; Luczak et al., 2006) and distributed memory storage (Hopfield and Tank, 1986; Leutgeb et al., 2005), both of which remain largely unexplored in part due to experimental constraints. Nevertheless, it is known that engaging neurons at an appropriate “population” level via patterned sensory input will immediately re-define their responses (Engert et al., 2002) and re-sculpt network organization over time (Sharma et al., 2000), suggesting a rich repertoire of processes that make networks responsive to patterned input.

Several methods have been developed which are effective to a certain extent in engaging neurons at multiple sites. Multi-patch clamp (Song et al., 2005; Be and Markram, 2006) offers precise control, but is incredibly laborious and restricted to only a few neurons at best. The mobile electrode can move and stimulate at different positions in slow sequence; alternatively, electricity has been elegantly routed to different locations in an electrode array by an experimenter one by one (Wagenaar and Potter, 2004; Sekirnjak et al., 2008), which is indeed the work that inspired the present study. However, the mobile electrode lacks the capacity to produce fast temporal patterns at multiple loci in parallel. Similarly, laser-guided stimulation (Fork, 1971; Farber and Grinvald, 1983), such as in glutamate uncaging (Callaway and Katz, 1993; Matsuzaki et al., 2008) or optogenetic stimulation (Petreanu et al., 2007; Wilson et al., 2012) has typically been applied to neurons sequentially which can only foster limited parallel dynamics due to limitations in beam speed and necessities in dwell time, not to mention requiring additional experimental steps such as the delivery of artificial transmitter and viral infection. Recent promising and technically ambitious work is emerging to mitigate limitations in beam speed (Shoham et al., 2005; Nikolenko et al., 2007; Grossman et al., 2010; Wilson et al., 2013). Similarly, advances in spatial light modulation (SLM) are allowing groups of cells to be targeted with precision, and multi-SLM methods (Carrillo-Reid et al., 2019; Marshel et al., 2019) are beginning to sequence or alternate the activation of small sets of these groups. Nevertheless, no system has been enacted for the simultaneous activation of more complex and rapidly changing patterns of neurons, or engaging plasticity in response to more multiplexed spatiotemporal control.

We reasoned that by distributing many individual electrodes on a fine scale throughout a brain network, we could then enact massively multi-site stimulation via 3 additional key components: 1) a software control system for rapidly designing and managing patterns, 2) analog electronics to actualize the electrical patterns and route them among an arbitrary number of electrodes, and 3) a digital gateway to mediate between the two and enforce exact temporal precision within the pattern. As described in the discussion, this configuration will likely be applicable to any many-channel stimulation system in the foreseeable future, though probably with ever-evolving actuators (electrodes, lasers, magnets, etc). We achieve one here using specific components that we believe offers a current best-in-breed combination of price, flexibility, and ease of use.

In building the system, we find that 1) a matrix-based programming language such as Python's scientific computing packages or MATLAB is ideal for the experimental expression of patterned stimulation, as it is centered around the manipulation of matrices, 2) an arbitrary number of stimulation channels can be controlled in parallel by applying time-division multiplexing, optical isolation and sample and hold technology, and 3) field programmable gate arrays (FPGAs) offer an inexpensive off-the-shelf way to flexibly link computers to real-time brain systems. Together these 3 components enable a “matrix stimulator,” allowing the experimenter to express complex stimulation protocols in arbitrary electrical patterns, delivered at sub-microsecond resolution across many channels to a brain circuit simultaneously.

To interface the stimulator to brain tissue, we made use of advances in multi-electrode arrays (Meister et al., 1991; Maher et al., 1999) that now enable measurements and perturbations of increasing sophistication (Eytan et al., 2003; Royer and Paré, 2003; Wagenaar et al., 2006; Chao et al., 2008; Johnson et al., 2010; Hottowy et al., 2012; Elyahoodayan et al., 2019) and where activation currents can be deployed by connecting the electrodes to custom control circuits (Regehr et al., 1989; Jimbo et al., 2003; Wagenaar and Potter, 2004). By routing our fast activation patterns through the many spatial channels of these arrays, we found that they could be used to drive patterned activity and explore new plasticity paradigms as originally proposed (Heck, 1995).

In the work presented here, we debut the system in acute slices of mouse visual cortex, demonstrating patterned generation of precise and robust activity throughout a cortical network, spatiotemporal activation of synaptic transmission converging on identified neurons, and potentiation of transmission across independent pathways. In turn, having control over distinct neurons and their output pathways now allows us to activate neurons in different timing sequences to directly probe novel paradigms of plasticity. As a proof of principle, we present experiments using the system to successfully drive multi-neuronal plasticity using spatiotemporal and sequential programming regimes across the cortex. Finally, we present a means by which multi-site stimulation technology can be meaningfully coupled with population recording, afforded by multi-neuronal calcium imaging, to provide a high-throughput and automated “network view” of both function and plasticity.

We thus offer a framework for the robust real-time patterned activation of many brain circuit locations from a standard computer. In the spirit of open source science, the system can be completely replicated by 1) downloading some freely available, and modifiable code and 2) mail-ordering a circuit from a freely available schematic. While we have demonstrated its straightforward application in brain slices from mice, the same system can be readily connected to a variety of electrode probes to unlock investigations of network plasticity in a range of neuronal circuits, animal models, and prosthetic applications.

## Materials and Methods

See also supplementary documents “Open Hardware and Software Components” for ordering details, “Technical System Detail” for deeper implementation notes, “Steps Toward Automated Experiments and Analysis” for integration with experiments and analysis, and “System Limitations and Future Opportunities” for potential modifications.

### “Matrix” Stimulator for Parallel Delivery of Spatiotemporal Patterns

The fundamental challenge in distributed brain stimulation is to 1) design and orchestrate complex energy patterns, and 2) distribute that energy to the appropriate places at the appropriate times. We found that reliable spatiotemporal stimulation benefits from 3 components—software control for complex and composable stimulation, simple but principled stimulation electronics to quickly distribute the electricity to the right locations, and a real-time programmable digital intermediary between the two to enforce temporal precision and overcome the temporal jitter of standard computer operating systems. The specific parts that enable this synergy, which can be replicated almost entirely via download and mail-order, are described here (Supplementary Figure 1). The methodology is designed to inter-operate with any multi-electrode array, and indeed any set of effectors that can controlled via fast analog patterns, to open up those channels so that the experimenter can focus on the patterns themselves from their software of choice.

#### Software Control for Flexible Stimulation

The system is built for compatibility with standard software approaches and demonstrated using the powerful and versatile MATLAB programming environment (MathWorks, Natick, MA). The only software routines required to run the stimulator is 1) a “send” function that allows the experimenter's spatiotemporal pattern(s) to be sent to the stimulator's memory, 2) a “go” command to trigger release of the pattern(s) at specific times, and 3) a single USB driver that is provided with the FPGA module (described below) and adaptable to many common programming environments. Once these are in place, the experimenter is free to create any “stimulation matrix,” in MATLAB, Python, or otherwise, which is simply a list of “time/voltage/electrode #” tuples, for activating one or more electrodes at a given time with various voltage levels. The experimenter then simply passes a “stimulation matrix” as a variable to the stimulator's “send” function, calls “go,” and it will be delivered to the electrodes. The experiment is free to use all of their software environment's computational complexity for defining and managing patterns, and the stimulator commands can naturally be called from within the experimenter's acquisition or analysis scripts in MATLAB or other languages.

#### A Field-Programmable Gate Array (FPGA) Provides a Versatile Interface From Computer to Electronics

Once the user's stimulation matrix is sent from the software (see above), it passes via a high speed USB cable to the “interface module” that enforces the real-time delivery of the stimulation, and consists entirely of a single inexpensive part that can be ordered online and is a standard in university classrooms—the Xilinx Spartan III FPGA integration module from Opal Kelly (XEM-3001, Portland, OR). Once this module is connected to your USB port, the only other step is to use the software that comes with this module to download our. BIT file into the module's FPGA—this will provide the circuitry configuration needed for stimulation. Alternatively, the FPGA provides a programmable hardware layer that can be further tailored to the experimenter's needs.

#### Analog Electronics to Connect an Arbitrary Number of Stimulation Electrodes

A complete circuit diagram of our stimulator electronics is freely available upon request, and can be fabricated online by submitting the downloaded schematic and parts list to an online circuit fabrication service (ExpressPCB.com, etc). Fundamentally, electricity is distributed by the stimulator by using 1) a single digital-to-analog converter to produce a bipolar voltage waveform (Texas Instruments TLC7628, Digikey, Inc.) that contains the aggregate waveforms for all electrodes of interest, 2) 3-to-8 line decoder/demultiplexers to route components of the waveform to the appropriate output pins (Texas Instruments CD74HC237E, Digikey) 3) sample-and-holds that maintain the supplied voltage at each output pin until an updated voltage arrives (SMP18, Analog Electronics) and 4) a final-stage Op-Amp for signal conditioning on each channel (OP495, Analog Electronics). A few additional op-amps, or/nor gates, and voltage overrides are scattered throughout the circuit as described for further optimization, but these are the primary components. The outputs of each channel's sample and hold are then fed into a universally compatible I/O connector block as described in the following section, to be connected to the electrodes of interest. For further ordering information, see supplementary document, “Open Hardware and Software Components.”

#### Interfacing the Digital Module With the Analog Circuit

The analog circuit requires only 2 inputs, one of 8 high/low wires and the other of 6 high/low wires, that connect to a row of pins on the digital module described above. The 8 wires allow the digital module to specify an 8-bit instantaneous voltage to be generated by the digital/analog converter. Similarly, the 6 wires specify a 6-bit address for which pin will receive that voltage and maintain it using its sample and hold. The 8+6 wires, operating at MHz speeds, therefore allow the digital module to deploy a rapid series of voltages across 2^6^ or 64 electrodes. More electrodes can be added by simply adding an additional wire (7 wires = 2^7^ = 128 electrodes, etc) and making a slight modification to the code of the FPGA module. Finally, the analog circuit requires power lines for ±5 and ±12 V, which we got from a standard low-noise computer power supply (Power-One MAP55-4000, Digikey). The entire set of connections for the stimulator therefore consists of: power, a USB input, and the internal wire track between the digital module and the analog electronics.

### Connecting the Stimulator to Any Multi-Channel Probe of Interest

The system is intentionally designed to be agnostic to the configuration of electrodes or other effectors. The endpoint of the stimulator is a universally compatible I/O connector block (SCB-68, National Instruments) where one can use screw terminals to connect the stimulator outputs to another wire of choice. Connecting the stimulator to that box is another universal cable (68-pin SCSI, SHC68-68, National Instruments) which has many adaptors available for potential “plug and play” compatibility with a variety of existing interfaces. Compatibility with SCB-68 breakout boxes thus provides a modular architecture by which the stimulator can be swapped from one arbitrary probe type to another with a single plug.

The cable is then adapted to the multi-electrode probe of choice using a second I/O connector block, and for the experiments described here, wired to either an MEA-60 planar array (Multi-Channel Systems, Reutlingen, Germany) or an MED-64 (Panasonic/Alpha-MED, Berkeley, CA) via their associated commercial base units.

### Stimulation Patterns and Interplay With Other Software

Stimulation trains consisted of biphasic pulses, 0–4.5 V in each direction, typically of 1 ms duration. Complex spatiotemporal patterns of these pulses could be built up and saved for re-use by creating waveform templates in MATLAB, scaling these waveforms in time, duration, and amplitude, and assigning them to different sub-patterns and pins via matrix operations mediated by graphical interfaces. Concurrent data acquisition for electrophysiology and imaging, also mediated by coordinated graphical interfaces in MATLAB, allowed for the systematic and real-time mapping of stimulation patterns to responses as in **Figures 2**–**6**. Stimulation channels could also be used as high resolution TTL trigger lines to couple other devices (e.g., frame grabbers) tightly in time in synchrony with stimulation pulses.

### Preparation of Acute Cortical Slices

C57BL/6 juvenile mice (P10-P20, Taconic) were anesthetized via exposure to isoflurane and decapitated, with the brain rapidly removed and immersed in <4°C cutting solution (25 mM NaHCO_3_, 25 mM glucose, 7 mM MgSO_4_, 2.5 mM KCl, 1.25 mM NaH_2_PO_4_, 2-110 mM CaCl_2_, 11.6 mM Sodium L-ascorbate, and 3.1 mM Sodium pyruvic acid; adjusted to 300-310 mOsm and 7.3 pH and bubbled with 95% O_2_/5% CO_2_). Coronal sections (300 μm thick) were cut, also in cutting solution, using a Vibratome Series 1000 Sectioning System (Vibratome, Bannockburn, IL), and incubated in artificial cerebral spinal fluid at room temperature for the remainder of the experiment (ACSF, 127 mM NaCl, 25 mM NaHCO_3_, 2.5 mM KCl, 1.25 mM NaH_2_P0_4_, 25 mM glucose, 1 mM MgCl_2_, and 2 mM CaCl_2_; adjusted to 300–310 mOsm and 7.3 pH; bubbled with 95% O_2_/5% CO_2_). Slices were transferred to a planar electrode array (MCS) and recordings were taken from visually identified pyramidal neurons located in layer 2/3. The intracellular pipette solution contained 100 mM potassium gluconate, 20 mM KCl, 10 mM HEPES, 4 mM MgATP, 0.3 mM NaGTP, and 10 mM Na-phosphocreatine, and it was adjusted to 295 mOsm and 7.4 pH. All experiments involving animals were approved by the Massachusetts Institute of Technology's Committee on Animal Care.

### Dissociated Cultures

For Supplementary Figure 2B, multi-channel planar electrode arrays (MCS) were sterilized by soaking in 70% ethanol (30 min) and overnight exposure to ultraviolet light. The center of the probe was then covered with 50 μl matrigel (BD Biosciences, San Jose, CA) and incubated at 37°F for 90 min. A pipette was then used to remove all but a thin film of matrigel, and hippocampi were dissected from postnatal day 1 Sprague-Dawley rat pups and cultured using standard methods. After 14 days *in vitro*, cultured probes were removed from the incubator, and culture media was swapped with pipettes for Tyrode's solution containing 145 mM NaCl, 3 mM KCl, 10 mM HEPES, 10 mM glucose, 1.3 mM MgCl_2_, 1.3 mM CaCl_2_, and 1 μ*M* glycine, adjusted to 305–315 mOsm and 7.4 pH. All experiments involving animals were approved by the Massachusetts Institute of Technology's Committee on Animal Care.

### Whole Cell Recording (Intracellular Patch Clamp)

The cortical slice was placed on the microscope in a submerged bath chamber integrated with a planar electrode array (MCS), and fit with a custom perfusion system which supplied 95% O_2_/5% CO_2_ room temperature ACSF to maintain the health of the slice over the duration of the experiment (3–4 h). The slice was manually positioned on top of the array such that the maximal number of electrodes was covered by the cortical region of the slice. A small net was added on top of the slice to ensure a tight coupling with the electrodes and to prevent deviations in X-Y position relative to the electrodes. The slice was then visualized with a Plan-NEOFLUAR 2.5x lens (Zeiss) and individual cells with an Achroplan 40x water-immersion lens with infrared-DIC optics (Zeiss), both detected with a cooled CCD camera (QImaging, Surrey, BC) projecting to a video monitor. Images were captured at 1,392 x 1,040 pixels using QCapture Pro (QImaging). Experiments were driven by custom acquisition and real-time analysis software written in MATLAB using a Multiclamp 700B amplifier (Axon Instruments) connected to a BNC-2110 connector block and M-Series dual-channel acquisition card (National Instruments). Borosilicate pipettes (3–5 *MΩ*, WPI) were pulled using a Sutter P-80 puller (Sutter Instruments). Gigaseal and rupture was achieved and patch recordings were continuously verified for low levels of leak and series resistance, as a prerequisite for further data analysis. For each recording, a 5 mV test pulse was applied in voltage clamp 10 times to verify normal input and series resistance. In current clamp 10 pulses (500 ms, 40–140 pA in 10 pA increments) were applied to verify cellular excitability that was comparable to slices in more standard chambers. Similarly, spontaneous extracellular postsynaptic currents (EPSCs) were sampled at 10 kHz and low-pass filtered at 1 kHz under voltage clamp at −60 mV to ensure levels of network drive consistent with our other slice preparations.

### Electrophysiological Analysis

Analysis of action potentials and EPSCs was performed using a custom software package written in MATLAB, with all events detected according to automated thresholds, and each event blindly verified *post-hoc* by the experimenter. For further clarity on the EPSC detection, see Supplementary Figure 5, and for detailed analysis procedures, see supplementary document “Steps Toward Automated Experiments and Analysis,” “Further analysis detail” section.

### Evoked Spiking and Synaptic Transmission Maps

A neuron was patched in current clamp and spiking was evoked by applying 3 V test pulses (1 ms) to individual nearby electrodes of a multi-channel planar electrode array (MCS). Each electrode was then stimulated systematically with pulses ranging in amplitude from 2 to 4.5 V to determine the stimulation level required to make the cell spike, and this process was repeated across each electrode of the planar array, stimulating at 0.33 Hz. This gave rise to an “action potential map” of the stimulation sites capable of inducing spiking in the patched cell for each stimulus voltage, and we observed concentric maps expanding outward with stimulation intensity (**Figure 2E**). For confirmation, spiking was abolished by adding 1 μm tetrodotoxin (TTX, Tocris) to the ACSF (**Figure 2C**). A similar methodology was employed for synaptic transmission by stimulating neurons connected to the patched cell while recording under voltage clamp at –60 mV, and mapping the stimulation sites and intensities that resulted in successful EPSC transmission to the patched cell. EPSCs were verified by adding 5 μm 2,3-dihydroxy-6-nitro-7-sulfamoyl-benzo[f]quinoxaline-2,3-dione (NBQX, Sigma) to the ACSF (**Figure 3B**).

### Population Stimulation and Calcium Imaging

Prior to interface with the multi-electrode probe, slices were incubated for 30 min in ACSF, and then transferred onto a filter membrane suspended over ACSF containing 1 mM Oregon Green 488 BAPTA-1 AM (OGB1-AM, Molecular Probes) and 0.4% Pluronic F-127 in DMSO, where they were maintained for 45 min while bubbling 95% O_2_ and 5% CO_2_. After incubation, they were rinsed in fresh ACSF for at least 30 min before being transferred to the stimulation probe for imaging during perfusion of oxygenated ACSF. Population responses were imaged using a cooled fast-CCD camera (QImaging QICam) with a Plan-Neofluar objective (2.5x, 0.075 NA). Fluorescence was continuously excited using a metal halide lamp (EXFO X-cite 120Q) passed through a blue filter on a Zeiss Axioskop while imaging through a green filter. Images were continuously acquired at 2.5x, 10–20 Hz, using 4 x as above, and various 4 binning. Temporally precise CCD imaging was achieved using Streampix4 software (Norpix) driven by synchronization signals from our MATLAB stimulation software that controlled the patterned stimulation.

### Calcium Imaging Analysis

During the experiment, images were saved by Streampix 4 software as multi-frame TIF files and analyzed automatically in MATLAB for responses. Regions of interest (ROIs) were generated by our software around each electrode of interest, and the mean fluorescence for each ROI was tracked from frame to frame, resulting in a time-varying intensity signal for each ROI. After correction for lamp flicker noise and dye photo-bleaching, ΔF/F % (DFF) was calculated for each stimulation event by determining the peak of the post-stimulus fluorescence and the local corresponding baseline. Response maps for single stimuli (**Figures 6e,f**) were plotted by translating the evoked DFF for each event at each ROI into color, and interpolating between these values in two dimensions. Population maps (**Figures 6g,h**) were then obtained by aligning the response maps for stimulation at different locations according to the two-dimensional position of the stimulation electrode. For detailed analysis procedures, see supplementary document “Steps Toward Automated Experiments and Analysis,” “Further analysis detail” section.

### Spatiotemporal Plasticity Paradigms

#### Multi-Site Tetanic Stimulation

A postsynaptic P10 pyramidal neuron was patched in voltage clamp (V_*h*_ = −60 mV) as above, and various stimulation sites were tested (2–4 V) for the ability to evoke EPSCs of moderate amplitude (ideally <100 pA). Two such sites were identified (Input A and Input B). For Input A, baseline EPSCs were first evoked at 0.1 Hz to produce the baseline depicted in Supplementary Figure 3C. For tetanus, the patched cell was then released from voltage clamp (I = 0, and a tetanus pattern was administered (10 bursts of 4–7 pulses at 100 Hz, separated by 150 ms). After tetanus, another baseline of EPSCs was recorded in voltage clamp (V_*h*_ = −60mV, 0.1 Hz) to assess the extent of synaptic plasticity that has occurred. Once a site that exhibited plasticity was identified, the process was repeated on a second, distant site (Input B).

### Associative Capture

A P14 pyramidal neuron was patched, usually in layer 2/3 of visual cortex, and multiple sites were stimulated at various voltages (2-4 V) until two sites were identified on different sides of the patched neuron of interest that produced distinct baseline responses, one superthreshold (strong) and one subthreshold (weak), and both exhibiting non-immediate response onset to indicate the contribution of synaptic input (which were abolished with NBQX blockade after the experiment). The 2 inputs (strong and weak) were then quantified in isolation before pairing (5–7 responses at each time point, evoked at 1 Hz), where the efficacy of the input was specified as the % of stimuli that evoked superthreshold responses in the patched neuron. Pairing (**Figure 4**, arrows) was then induced by stimulating the strong input followed by the weak input, with an interval of 15 ms between the stimuli, and repeating the pairing 100 times at 0.2 Hz. After pairing, response quantification resumed, and was repeated at multiple intervals in different experiments as shown in **Figure 4**. Association of the weak input usually occurred within 30 min of the pairing interval, with the weak input transforming to 100% efficacy in all experiments irrespective of the location of stimulus sites.

### “Moving Bar” Fast Sequential Stimulation

Responses were measured as described for either single cells (**Figure 5**) or regional populations (Supplementary Figure 2), while a “moving bar” stimulus was delivered to the system. In this stimulus, all electrodes in a given column were activated simultaneously, and the activated column was “swept” from left to right, at 100 ms (Supplementary Figure 2) or 80 ms between columns (**Figure 5**) where the first column of electrodes was activated, followed by the second column 80 or 100 ms later, and so on until all 8 columns had been activated. over a period of 640 or 800 ms. This moving bar activation pattern was repeated every 10 s, for 100 repetitions. Activation for a given electrode within the column involved a 1 ms biphasic pulse of 3–4.5 V amplitude delivered to that electrode, and activation pulses were delivered to all electrodes in a column in synchrony.

### Sequential Stimulation During Population Imaging

Population responses were monitored across multiple rows and columns of electrodes to provide dozens of response sites, while each response site was stimulated in sequence (4–4.5 V per pin, 5s interval between stimuli) from left to right across each row and then repeating on the next row down. In some experiments each pin was stimulated only once, and sometimes 3 times each (5 s interval). The entire sequence of ROI electrodes was stimulated, and this was considered a “before training” population response map. During sequence training, this sequence pattern was then repeated 6 times without pause, and after some consolidation time (5–10 min) an “after training” map was acquired and compared to the first. This protocol of sequence training was compared to “unpatterned” training with the same number and intensity of training stimuli. Sequence training was also attempted in saturating conditions of the synaptic blockers NBQX (5 μ*M*), AP5 (10 μ*M*), and bicuculline (20 μ*M*), which were added to the slice manually prior to training, and then washed out for 60 min to ensure a clean retest. Finally, sequence training was performed in the presence of these synaptic blockers, blockers were rinsed out, and then training was performed a second time without the presence of the drugs, with the ratio of response levels post training compared to baseline ones (**Figures 6j,k**).

### Statistics

All statistical comparisons were performed with paired two-tailed *t*-tests. All means are reported with the standard error of the mean, unless otherwise stated.

## Results

### A Platform for the Patterned Stimulation of Neuronal Networks

#### Analog Electronics for an Arbitrary Number of Stimulation Electrodes

Any platform for spatiotemporal stimulation as a first step requires the control of an energy flux and the distribution of that flux. We found that an arbitrary number of stimulation electrodes could be controlled independently by means of a simple circuit (See Methods, Supplementary Figure 1). that would generate a time-varying voltage, distribute that voltage at kHz-Mhz speeds to different electrodes according to synchronized routing signals, and then “sample and hold” that voltage at each electrode until the next sub-millisecond update point. The essential enabling aspect of this multi-site control was time-division analog multiplexing, in which all electrodes- electrical waveforms are initially generated along a single communication channel as one “complex” intermingled waveform, thus necessitating only a single digital-to-analog converter, and circumventing a common limitation in computer interfaces. This complex waveform is designed to carry pieces of each electrode's voltage waveform at recurring intervals in the time domain, and this regularity can be exploited by synchronizing a cycling stream of address bits that rapidly activate each electrode channel to sequentially “open” and “grab” the next voltage during its allotted time slot, so that one cycle delivers new voltage values to all electrode channels at sub-microsecond speeds, and over time the complex waveform is distributed among an arbitrary number of subsidiary channels. These channels are then equipped with two further properties: 1) a “sample and hold” component to maintain the previous voltage value over time until the next voltage update is received, so that the voltage does not fall toward zero in the small time gap between updates, and 2) optical isolation from the rest of the stimulator circuit so that each channel's waveform is not disrupted by the “loud” digital address electronics, or by the waveforms of other channels, so that truly independent stimulation can be driven across all channels in parallel.

#### Software Control for Flexible Stimulation

Foundational stimulators with more than one channel, such as the original Master-8 (A.M.P.I.), relied on sequences to be entered manually from a console on the device. We found that patterned orchestration across many channels necessitated high-level PC programming to interchangeably create timing loops, select groups of channels, store electrode maps, chain different patterns into more elaborate sequences, and consolidate with data acquisition. An ideal environment for such computations is a matrix-first programming environment such as MATLAB or Python/Numpy/Scipy/Pandas, which are built around manipulating matrices of data and thus provided a natural fit for representing and updating spatial arrays over time. As MATLAB is a common language for neuroscience analysis (and increasingly acquisition), we reasoned that an interface between this language and multi-site stimulation would be useful to many laboratories irrespective of their application. We developed two core MATLAB scripts that can be incorporated into the user's own software, available upon request – one for downloading an arbitrary spatiotemporal stimulation pattern to the chip, and the other for triggering its release.

#### A Field-Programmable Gate Array Provides a Versatile Interface From Computer to Electronics

We wished for our stimulator setup to work with any lab computer. One thing all modern computers have in common is a USB (universal serial bus) port. However, instructions from the USB port cannot directly drive stimulation because they are too erratic in time. Instead we needed a “one-piece” onboard mini-computer to provide a computational intermediary between the USB port and the electronics described above. We found an inexpensive off-the-shelf module (Opal Kelly XEM3001, with Xilinx Spartan-3) that 1) has a USB connector and drivers for MATLAB, 2) has sufficient RAM to hold downloaded stimulation waveforms prior to release, and 3) is based on field-programmable gate array (FPGA) technology, meaning that we could dynamically “re-program” the electronic circuit for different uses without any rewiring, and other labs could simply “download” our circuit onto their purchased module. This module can therefore receive patterns over USB, store them in memory, and dispatch them upon a received command in precisely timed sequences. This means that anyone could buy this inexpensive piece, download our circuit pattern, and they will have their interface from MATLAB to a many-channel stimulator.

**Figure 1:**
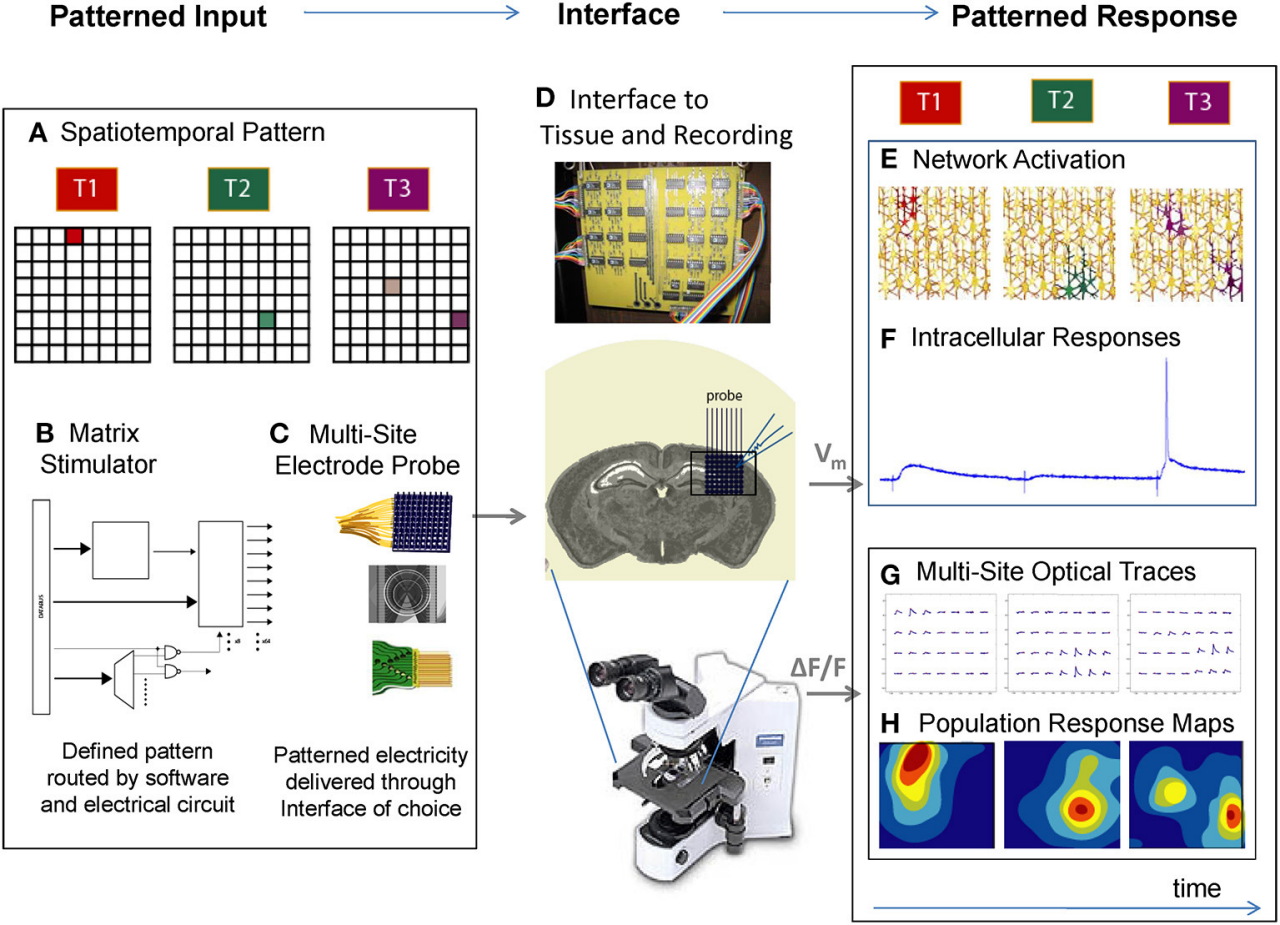
A platform for the patterned stimulation of neuronal networks. **(A)** The experimenter uses graphical interfaces or programmed scripts to define a spatiotemporal pattern in a software matrix, such as activating one spot at time 1 (T1), another spot at time 2 (T2), and 2 different spots at time 3 (T3). **(B)** Using open protocols and rapid signal processing, a custom “matrix stimulator” generates the electrical waveforms and routes them in real time it to the corresponding electrodes. **(C)** The experimenter selects a 2D or 3D electrode array of choice, and activates the corresponding software lookup table. Any electrode array can be connected to the stimulator via a breakout box. **(D)** The stimulator's electrodes are interfaced to the brain area of interest, *in vitro* or *in vivo*, and simultaneous recording can be taken via electrophysiology or imaging. **(E)** Playing the spatiotemporal pattern defined in **(A)** results in neuronal activation at discrete locations of the network. **(F)** Single-cell measurements during this activation would yield a variety of synaptic and spiking responses from the cell of interest. **(G)** Multi-site “population” responses during this activation could in turn yield a “network view” of distributed activation **(H)** as the spatiotemporal pattern is delivered. In this way, multiple neurons can be activated at different spatial locations, in arbitrary temporal patterns.

As a result, the entire patterned stimulation process is transparent to the experimenter, who is free to flexibly select patterns (or sequences of patterns) according to the experiment at hand, and push the button to orchestrate them in synchrony with electrophysiology or high-speed imaging (Figure 1). In practice, the experimenter might use either graphical interfaces or programmed scripts to define a spatiotemporal pattern from a software matrix (Figure 1A). Then, the pattern is sent to the stimulator and triggered for release through the circuit (Figure 1B, Supplementary Figure 1). The stimulator in turn connects to a universal “connector box” (see Methods) where the stimulation channels are mapped to any cable or probe of the experimenter's choosing, including fine-wire grids, 3D arrays, or planar arrays (Figure 1C). The multi-electrode interface is then placed against the brain area of interest (Figure 1D), and the resulting distributed (direct or synaptic) neuronal responses (Figure 1E) can be assayed at either the single cell level (Figure 1F) or the network level (Figure 1G). Pairing stimulation with whole cell recording gives us a precise, single-cell resolution readout of the response to multi-site stimulation, allowing us to further investigate synaptic activity, while population imaging of calcium responses informs us on distributed response behavior across the network (Figure 1H). The stimulator is designed to introduce no noise into these measurements with the exception of the electrical stimulus artifact itself.

### Versatility of Spatiotemporal Activation Patterns and Functional Interface

The system can in principle be applied at multiple scales depending on the effector, from electrodes distributed throughout an animal, to a micro-clustering about a dendritic arborization. In the present work we connected ours to planar arrays of electrodes (200 μ*m* spacing) interfaced to acute slices of mouse visual cortex, which routinely remained functionally interfaced for 10 h (Supplementary Figure 2A). From the very first characterizations we found that spatial stimulation could be applied in diverse contexts (Supplementary Figure 2), including different electrode geometries (size, spacing and pattern), various tissue preparations (acute slice and dissociated cultures), and distinctive activation paradigms (i.e., complex spatial patterns and “natural” stimuli) where applied patterns resulted in immediately discernible spatiotemporal responses in the tissue as visualized by calcium imaging. We also could use the system to produce waveforms of arbitrary shape, not just square pulses (Supplementary Figure 4). What follows are detailed characterizations and extensions of these initial validations.

### Precise Spiking Activity Evoked Across Neurons

**Figure 2:**
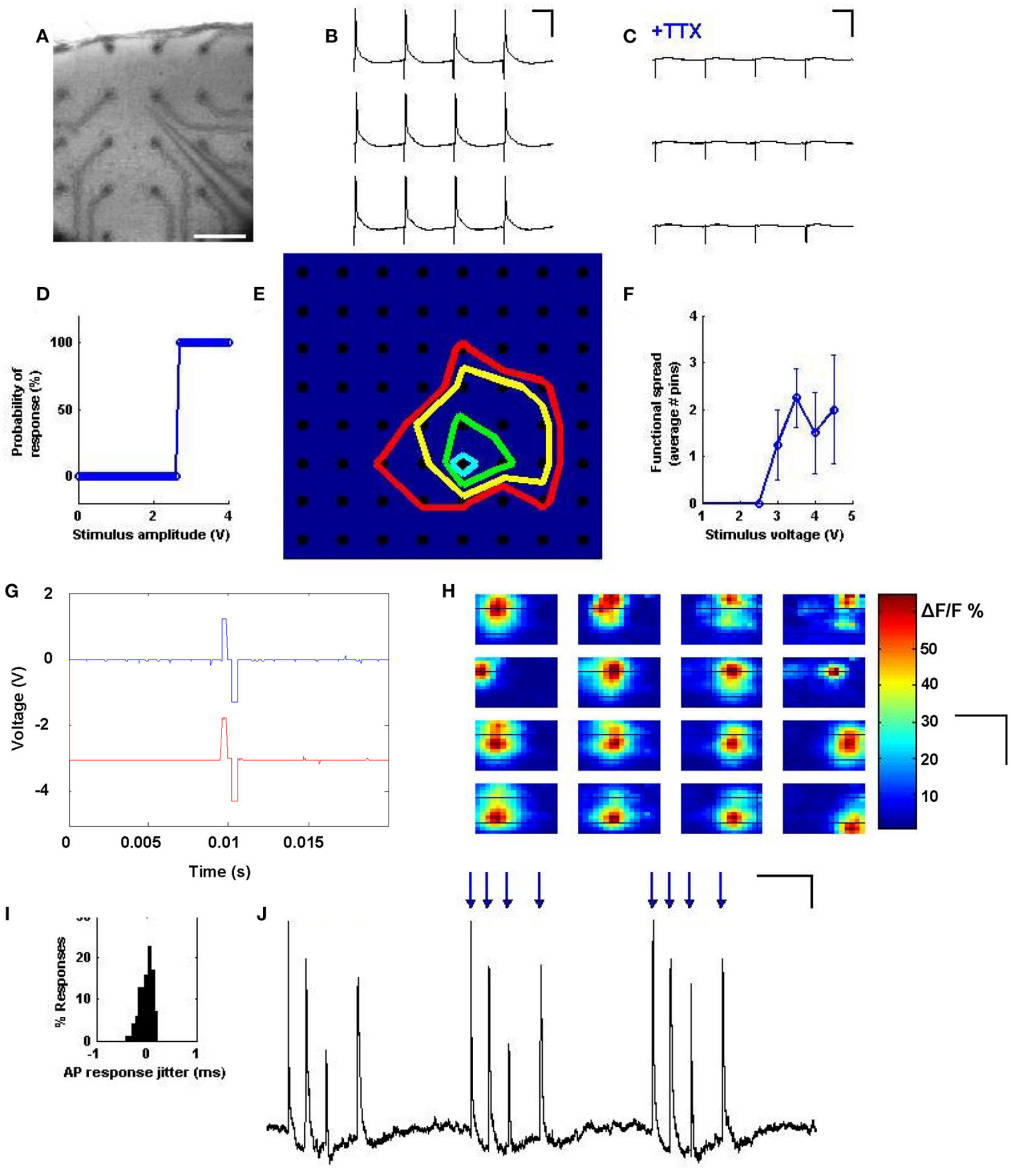
Precise spiking activity evoked across neurons. **(A)** A cortical slice is interfaced with electrodes from the stimulator, and simultaneous patch-clamp is achieved on a layer 2/3 pyramidal cell, as visualized at 2.5x. Scale bars: 200 μm. **(B)** Stimulating a pin during current clamp near the patched cell results in a single action potential for every stimulus pulse. Scale bars: 50 ms and 30 mV. **(C)** Responses are abolished with 1 μM TTX. Scale bars: 50 ms and 30 mV. **(D)** Probability of eliciting an action potential exhibits an all or none behavior with stimulus intensity, such that past a certain voltage, responses are 100% reliable. **(E)** Map of spatial precision with increasing stimulus intensity. A cell was patched at row 6, column 5, and colored lines were drawn to delineate the electrodes that are capable of exciting that cell at increasing voltages of 2V (blue), 3V (green), 3.5V (yellow) and 4V (red). **(F)** Average effective range of the stimulus as a function of stimulus intensity. For each data point, *N* = 64 pins were stimulated, and loci resulting in a spike were taken and their distance from the patched cell averaged. **(G)** Temporal precision: individual stimulus waveforms can be generated with sub-millisecond precision, and across channels distinct waveforms can be simultaneously generated with tight temporal coordination. **(H)** Spatial mapping of stimulus and response can be assayed by using population calcium imaging to visualize the spread of activation when each site was stimulated. Shown are 16 response maps taken while stimulating one electrode at a time, at maximal voltage, in a 4x4 grid, with the map position corresponding to electrode position. Responses within the map seem to reflect the position of the stimulation site. Scale bars: 500 μm, 800 μm. **(I)** Responses evoked by the stimulator always occur within <1 ms of each other, indicating temporal precision across trials. **(J)** Temporal patterns of stimuli (blue arrows) can be used to reliably drive arbitrary spike trains. Scale bars: 150 ms and 4 mV.

In order to show that multi-site stimulation could reliably evoke single action potentials, a neuron was patched in current clamp and 3 V pulses were applied to electrodes of the planar array which were near to the patched cell (Figure 2A). We observed single action potential spikes (Figure 2B) which were abolished in the presence of 1 μM TTX (Figure 2C). We next examined the threshold behavior of the evoked responses by stimulating an electrode near the patched cell with pulses increasing in amplitude from 2 to 4.5 V; this indeed revealed a threshold activation required to induce an action potential (Figure 2D). In a similar setup, all electrodes across the planar array were stimulated with increasing voltage in order to determine which electrodes could be stimulated to elicit a response from the patched cell. At each stimulation intensity, a specific population of electrodes within a certain radius from the patched cell evoked action potentials, suggesting a correlation between population radius and voltage amplitude (Figures 2E,F).

Bidirectional and distinct stimuli could be produced on multiple channels with tight temporal registration (Figure 2G). The locational correspondence and spatial extent of the activation spread was further characterized by stimulating electrodes under a cortical slice bulk loaded with the calcium indicator Oregon Green BAPTA 1-AM (Figure 2H). Here, simultaneous population imaging at 2.5x shows activation of distinct locations depending on which electrode is stimulated. We also measured the precision of the effective stimulation in the time domain by quantifying time variance between stimulation and action potential response (Figure 2I), which exhibited sub-millisecond precision. A detailed analysis of the time resolution of our system (see Supplementary document, “Analysis of Temporal Precision”) suggests that this minimal jitter is likely biological in nature. Precision at driving repeatable timing patterns was also evaluated by stimulating an electrode near the patched cell in a random computer-generated temporal pattern, and comparing the activity of the patched cell to the random pattern applied (Figure 2J). These results indicate a precise spatial and temporal control over evoked spiking using the spatiotemporal stimulator.

### Synaptic Transmission via Multi-Site Stimulation

**Figure 3:**
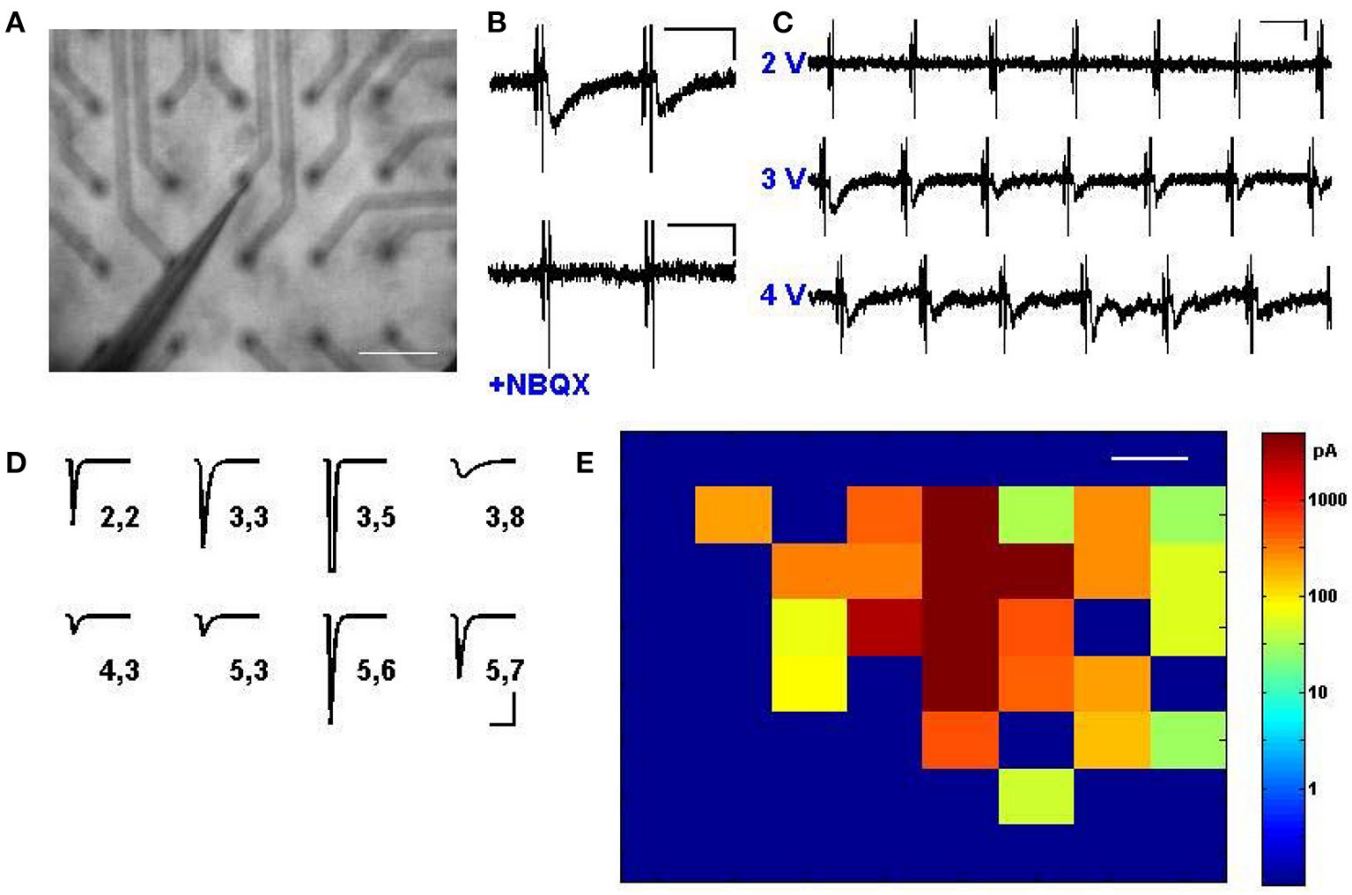
Synaptic transmission via multi-site stimulation. **(A)** A cortical slice is interfaced with electrodes from the stimulator, and a cell is patched in voltage clamp near the middle of the electrode array, as visualized at 2.5x. Scale bar: 200 μm. **(B)** Excitatory postsynaptic currents (EPSCs) can be elicited by stimulating electrodes distant from the patched cell (top) and responses are abolished via application of NBQX to block AMPA receptors (bottom). Scale bars: 80 ms and 50 pA. **(C)** Synaptic transmission is engaged to different degrees depending on stimulation intensity, and exhibits an “all or none” property characteristic of minimal stimulation. Scale bars: 80 ms and 50 pA. **(D)** A rich array of EPSCs, presumably arising from distinct subpopulations of cells, can be evoked by activating different stimulus locations. Shown is an automated map where numbers correspond to the row and column of the pin location used to excite a single postsynaptic cell, and average evoked EPSCs are shown, including one with direct activation at electrode (3,5) which led to the transient escape from voltage clamp. Scale bars: 80 ms and 30 pA. **(E)** Mapped visualization of distinct EPSCs elicited by stimulation of every pin in the array at 4.5 V, indicating heterogeneous responses from across the cortex. Color bar indicates response amplitude; the patched cell was located in the middle of the red at row 3, column 5. Scale bars: 200 μm.

**Figure 4:**
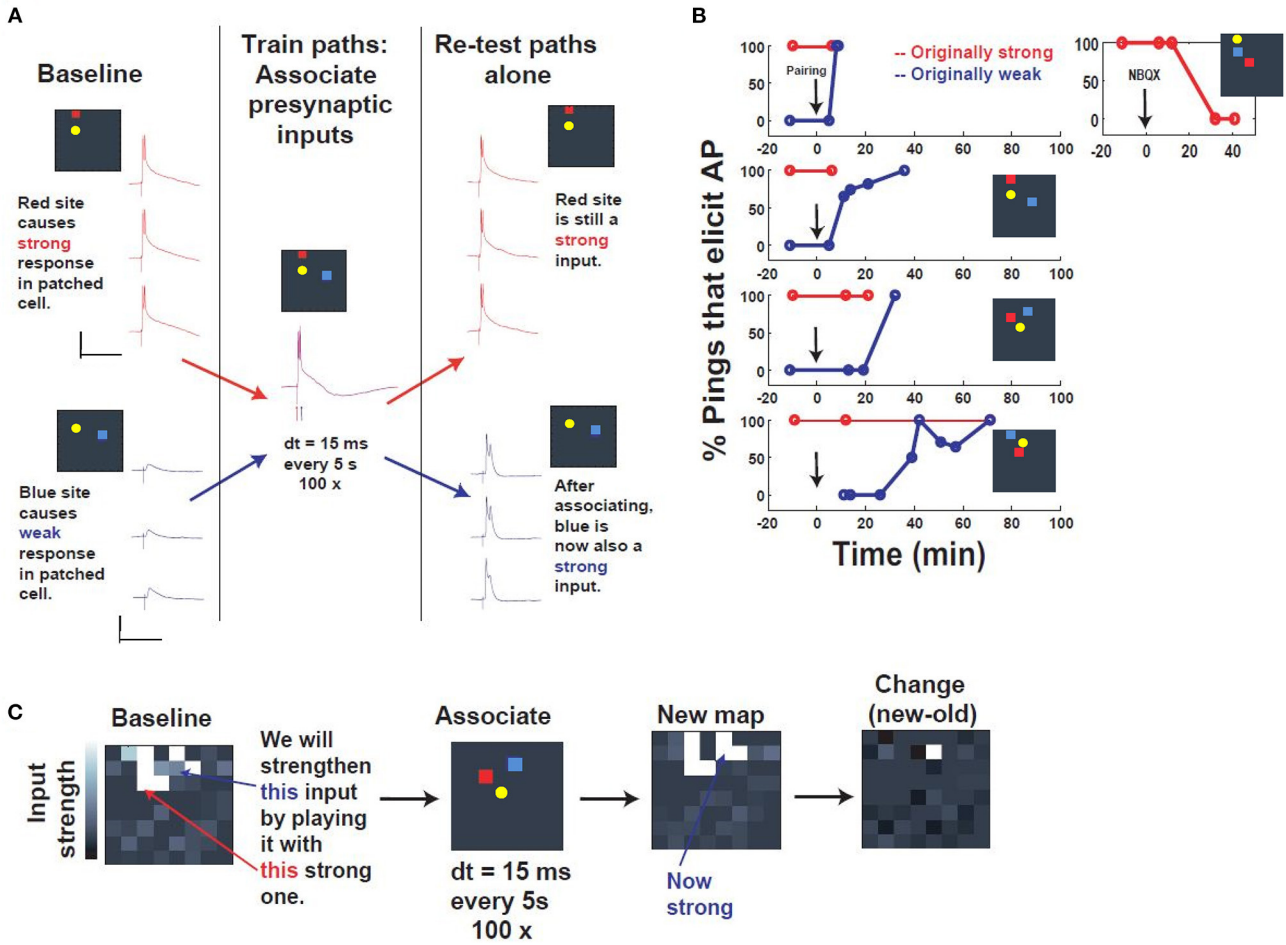
Associative capture of synaptic strength via spatiotemporal programming. **(A)** Description of the training. First, a “postsynaptic” cell was patched in current clamp, and presynaptic inputs were searched by pinging different electrodes, until two distinct presynaptic input sites were identified that could activate the patched cell (yellow) in distinct ways: a red “strong” site that could drive the patched cell to fire, and a blue “weak” site that elicited a subthreshold response. These 2 input sites were then “associated” by stimulating them within 15 ms of one another, red before blue, every 5 s, 100 times. Re-testing after association revealed that, while the strong site still elicited an identical response, the weak site now was substantially strengthened, such that it could drive the cell as well. Scale bars: 100 ms, 30 mV. **(B)** Repetition in multiple slice experiments and timecourse. Plotted is the input strength (% pulses that elicit a postsynaptic AP) of 2 distinct input sites (red, “originally strong,” or blue, “originally weak”), which start at 100 and 0%, respectively. After the associative pairing (arrow), the “originally weak” input begins to change in input strength, until it eventually reaches 100% efficacy. Inset: NBQX application abolishes the strength of the strong input, verifying that these inputs are synaptic. **(C)** “Network view” of all inputs to the patched cell, before (“Baseline”) and after assocation (“New map”). Strikingly, the change in the map (“Change”) reveals that most of the change is concentrated in the specific input that was associated with the strong input during the associative programming.

**Figure 5:**
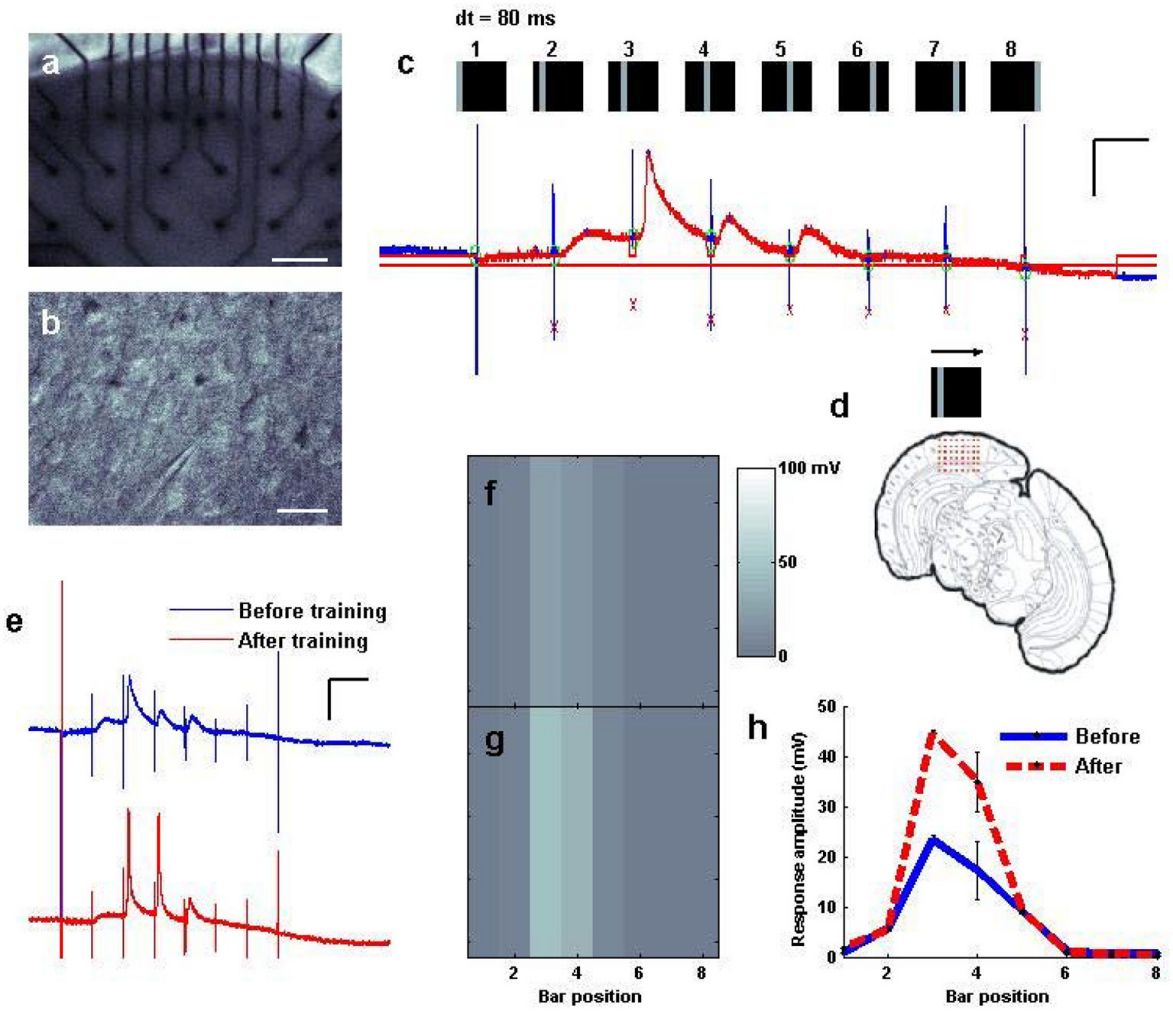
Fast “moving bar” entrainment, by sequential activation of nearby cortical regions. **(a)** A cortical slice was interfaced with an electrode array, as visualized at 2.5x. Scale bar: 200 μ*m*. **(b)** An intracellular recording was established with a pyramidal cell. Scale bar: 50 μ*m*. **(c)** A whole cell recording of the patched cell while a “moving bar” stimulus of 3.5 V per pin was delivered to consecutive columns of electrodes sweeping across the cortex from left to right at 80 ms intervals between columns. Stimulus artifacts are tagged in blue, where the stimulus was delivered at each bar position; responses to each are tagged in red. Scale bars: 60 ms, 20 mV. **(d)** Schematic of the electrodes relative to the coronal slice. **(e)** “Training” of the bar was undertaken by sweeping the bar 100 times every 5 s. Response to the bar is measured before and after training. Scale bars: 100 ms, 20 mV. **(f,g)** Mapped visualization of response to the stimulus before **(f,g)** training repetition. **(h)** Average amplitude of responses at each location of the map captured either before (*N* = 10 sweeps) and after (*N* = 15 sweeps) repetition training.

Given that multi-site stimulation could be used to selectively activate neurons in distinct locations (Figure 2), we next evaluated whether the activation of these neurons could be used to drive multiple synaptic inputs that targeted distant cortical zones or neurons of interest. We thus replicated the setup of Figure 2, but patched neurons in voltage clamp at –60 mV while driving electrodes in the areas around it sequentially to evoke input drive from various sites (Figure 3A). From our very first experimental attempt it was straightforward to identify electrode sites and stimulus strengths that drove reliable AMPA receptor-mediated transmission (Figure 3B), and exhibited characteristic properties of short-term depression and paired-pulse facilitation (data not shown). These evoked transients also exhibited an “all-or-none” behavior with increasing voltage (Figure 3C), suggesting that a threshold activation was required to engage the firing of the presynaptic neurons responsible for the synaptic drive. Stimulating different locations in the electrode array at various distances from the patched neuron resulted in a wealth of heterogeneous synaptic responses (Figure 3D), which included weak synaptic responses, strong synaptic responses, direct neuronal activation, and no detectable responses, in a manner that corresponded anatomically to the location of the patched postsynaptic cell (Figure 3E). These evoked inputs were physiologically independent, as verified by their functional summation and independent responsiveness to activity histories (see also Figures 4, 5 and Supplementary Figure 3). With the ability to measure synaptic responses while routinely engaging multiple activation sites, we thus have a platform by which to compare and combine multiple synaptic inputs in time onto identified postsynaptic cells of interest.

### Associative Capture of Synaptic Strength via Spatiotemporal Programming

Following verification that canonical synaptic plasticity could be achieved routinely and sequentially via tetanic stimulation in independent sites (Supplementary Figure 3), we evaluated whether the spatiotemporal stimulator could be used to selectively associate multiple entrained pathways to the same neuron. The ease of use and versatility of the stimulator's software-programmable electrodes allowed us to rapidly seek out convergent synapses, and then search for patterns capable of driving such coordinated plasticity between sites. In the paradigm that we arrived at, we took a P14 neuron that had been patched, and stimulated spatially disparate electrodes with increasing voltage until two sites were identified that produced distinct baseline responses, one superthreshold and one subthreshold (denoted as “strong” and “weak,” respectively, in Figure 4). These responses also exhibited non-immediate response onset, suggesting the contribution of synaptic input, which was further validated by NBQX wash-in (Figure 4B). The goal of this experiment was to evaluate the strength of two inputs before and after a novel potentiation pattern deliverable to arbitrary pairs of inputs via our spatiotemporal stimulator (Figure 4A). The baseline response was defined as % stimuli which evoked superthreshold responses in the patched neuron, quantified in isolation from 5 to 7 responses evoked at 1 Hz (Figure 4B).

The potentiation pattern we used was derived from demonstrations of associative synaptic plasticity in visual cortex (Frégnac et al., 1988; see also Discussion), and was a close temporal pairing, stimulating the strong input followed by the weak input, separated by a 15 ms interval and repeated 100 times at 0.2 Hz (Figure 4A). Responses were sampled at various time intervals after the pairing paradigm and indicated association of the weak input to 100% efficacy within approximately 30 min of pairing. These results were consistent across all experiments, independent of input location (Figure 4B). Comparison of all electrode responses before and after pairing also shows that potentiation is selective and distinct to the chosen “weak” input in the training paradigm (Figure 4C). The accessibility of the system thus allowed us to identify and apply a novel spatiotemporal pattern of stimulation for associative synaptic plasticity.

### Fast “Moving Bar” Entrainment, by Sequential Activation of Nearby Cortical Regions

Realizing that we could use the system to drive plasticity involving either a single input (Supplementary Figure 3) or interactions between inputs (Figure 4), we wished to evaluate whether our stimulator could produce electrical activation in fast spatial sequences to be delivered to the cortical slice, whether those fast sequences could drive distinct sequences of responses in recorded neurons, and whether these patterns could be adjusted to drive plasticity in the resulting responses. A cortical slice was therefore again interfaced with a multi-electrode probe (Figure 5a) and a pyramidal cell patched (Figure 5b). We used the stimulator to activate columns of electrodes one by one every 80 ms in a sweeping pattern from left to right, resembling a “moving bar” (Engert et al., 2002) of sequential activation (see also Supplementary Figures 2D,E). Feedback from the patched neuron in current clamp indicated appropriately timed stimulus artifacts (Figure 5c, tall blue lines), some of which were followed by depolarization of the neuron to degrees that were different for each location in the sequence (Figure 5c, red regions). Automatic detection algorithms could monitor these responses, map them to their input locations, and identify changes over time, thus facilitating the search for effective plasticity parameters. This sequential activation, sweeping across the slice with a period of 640 ms, was then run repeatedly (training; Figure 5d) every 5 s for up to 100 times (5–10 min). The response to the pattern after training was then plotted in comparison to the response before training, which in the case of this pattern revealed a noticeable strengthening of total responsiveness and an increase in the likelihood of neuronal spiking (Figures 5e,f vs. Figure 5g). Significant differences in responsiveness before and after training were observed for this pattern (Figure 5h) in contrast to various other fast spatial patterns attempted, including random squares, and random bars of comparable duration (data not shown), and potentiation using this pattern was replicated on 3 distinct slices. Thus, it is possible to use fast spatiotemporal stimulation to drive distinct sequences of responses in a neuron of interest, and change those responses over time as a result of methodical re-activation (see also Supplementary Figure 6).

### An Assay for Population Recording During Population Stimulation, and Sequential Entrainment of Optical Responses

**Figure 6:**
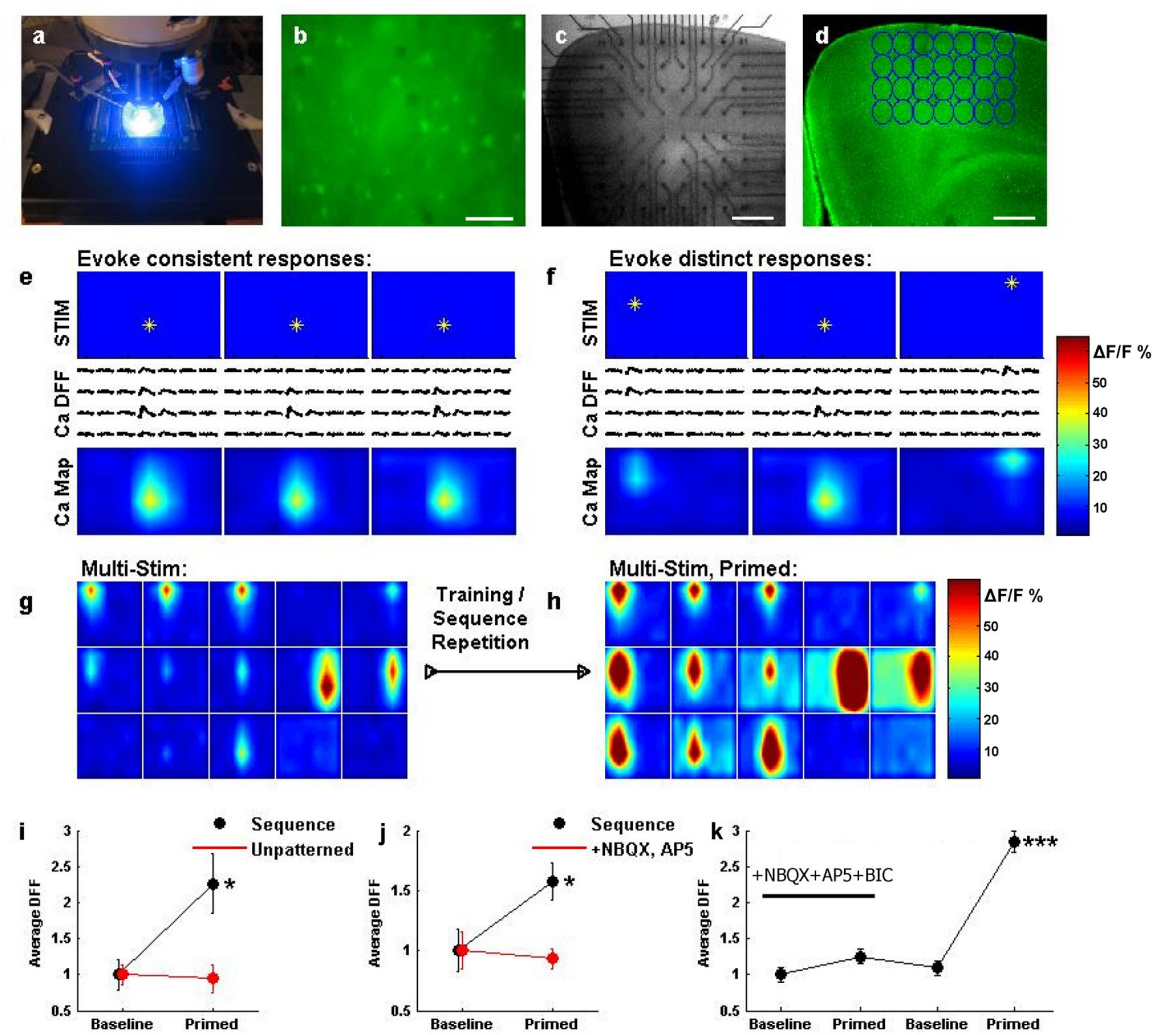
An assay for network recording during population stimulation, and sequential entrainment of optical responses. **(a)** A system view, illuminating fluorescence using 488 nm light during fast CCD imaging. **(b)** Layer 2/3 of a cortical slice bulk loaded with Oregon Green BAPTA 1-AM, as visualized at 40x. Scale bar: 100 μ*m*. **(c)** Cortical slice interfaced with electrodes from the stimulator, as visualized at 2.5x for experimentation. Scale bar: 400 μ*m*. **(d)** Cortical slice from **(c)** has been bulk loaded. Regions of interest (ROIs, blue circles) are selected around the location of each electrode, to quantify the calcium response during stimulation. Scale bar: 400 μ*m*. **(e)** Stimulating an electrode (top row, star) results in a change in calcium fluorescence that closely matches the spatial area of stimulation (middle row, traces). Plotting the response amplitude in color (bottom row, color) depicts the network activation map in response to the stimulus. Repeating this stimulus 3 times results in very similar network activation maps. **(f)** Stimulating 3 different electrodes results in distinct response traces and network activation maps, but the responses follow the stimulated electrode. Scale bars: 100 ms and 50% dF/F. **(g)** Sequential activation of multiple locations. Each box represents a network activation map (as in **e**) resulting from a single stimulus. The top row of boxes shows activation maps for electrodes in the top row of the array, from left to right, etc. Responses were taken while sequentially stimulating each electrode, from left to right across each row, for each row. **(h)** The sequence stimulation described in **(g)** was repeated several times, and this patterned activation seemed to induce an awakened state for the network. Most ROIs showed larger responses after training, and some ROIs even show responses where there previously were none. **(i)** Network activation (*N* = 28 ROIs averaged for each data point in **i–k**) was significantly potentiated following sequence training when comparing the first sequence activation (black, baseline) to a sequence after training (black, primed, *P < 0.05). Potentiation did not occur if comparable levels of unpatterned stimulation were delivered (red). **(j)** Repeating sequence training on a different slice again yielded potentiation (black, *P < 0.05), but doing so in a slice in the presence of synaptic blockers prevented potentiation (red). **(k)** In a different experiment, adding synaptic blockers prevented potentiation, but then washing those blockers out and repeating sequence training resulted in significant potentiation across the network responses (***P < 0.001).

The nature of our stimulation system opens the door for the routine testing of network responsiveness in a wide variety of brain areas, stimulation patterns, drug conditions, and plasticity statuses. The promise of such a system can be fully realized with the introduction of an automated method for measuring responsiveness, as opposed to the non-automatic patch clamp used for its high precision in first validations of the system (Figures 2–5). We therefore developed the ability to automatically detect and quantify calcium imaging transients during multi-site stimulation, across the network at low (2.5x) magnification. The current system integrated a cooled-CCD camera and arc lamp excitation (Figure 6a), but there is no reason why it would not be compatible with two-photon population imaging *in vitro* or *in vivo*. Bulk loading of acute slices with Oregon Green BAPTA-1 AM led to discernible baseline loading of populations of neurons throughout the slice (Figure 6b). The slice was then brought into apposition of the electrode interface (Figure 6c) and regions of interest were designated through which to track changes in fluorescence during patterned stimulation (Figure 6d). First, we stimulated a single location in the network (Figure 6e), and observed a localized calcium response in an area centered at the electrode location. Stimulating different locations (Figure 6f) resulted in a calcium response that followed the stimulation position.

We therefore had the means to stimulate sequential patterns while observing the consequent responses, or changes in those responses during stimulation. We attempted a “multi-stim” sequential activation of the cortical network from left to right, on each sequential row and observed matching responses across the network (Figure 6g). Interestingly, and to our surprise, replaying this sequence repetitively led to an incredible potentiation of measured population responses (Figure 6h). This was not simply due to repetitive activation, as a similar potentiation was not observed following comparable “unpatterned” stimulation (the same number and intensity of stimuli applied to randomly selected pins, Figure 6i) or the same total amount of stimulation delivered repeatedly to single locations (data not shown), suggesting that networks may be sensitive to the patterned activation of multiple cortical locations. This potentiation was also observed to depend upon synaptic transmission between neurons, as repeating the protocol led to successful potentiation with normal synaptic transmission, but no potentiation in the presence of the excitatory transmission blockers NBQX and AP5 (Figure 6j). Furthermore, repeating the sequential activation in the presence of NBQX, AP5, and the inhhibitory transmission blocker bicuculline prevented potentiation in response to the sequential stimulation, while washing out these drugs in the same slice and then repeating sequential stimulation produced a robust (perhaps even stronger) potentiation (Figure 6k).

Thus, our system may in principle be used to disclose how networks are sensitive to patterns, what those patterns are, and the molecular pathways that enable this sensitivity, as revealed via a form of high-throughput network interrogation.

## Discussion

### A Method for Driving the Patterned Interplay of Populations of Neurons

We made use of fine micro-electrode technology (Meister et al., 1991; Maher et al., 1999; Donoghue, 2002; Jimbo et al., 2003; Nicolelis, 2003; Wagenaar and Potter, 2004) to enable robust and independent communication channels at a small scale appropriate for network activation. Connecting such a system to a custom, 64-channel programmable stimulator that we built enabled the tissue to then be stimulated in fine patterns, allowing us to activate neurons (Figure 2), evoke synaptic transmission (Figure 3), and engage groups of cells in distributed forms of plasticity (Figures 4–6). Having fast, reliable stimulation patterns allows us to trigger multi-neuronal plasticity for the first time without requiring the laborious use of multiple patch pipettes (Be and Markram, 2006). Finally, we combined our patterned stimulation with network-scale recording (Figure 6) to detect changes in distributed responses in response to distributed activation patterns.

This technique does not rely on chemical or genetic effectors, and as such it can easily be applied to different tissue environments (Supplementary Figure 2). Furthermore, the principles we have established are relevant beyond the specifics of the system and compatible with many familiar probe technologies already in use, allowing the system to be adapted to different electrode array geometries and spatial scales (Supplementary Figure 2). At the same time, rather than providing cell-specific stimulation, electrodes allow us to study synaptic interactions in a manner which inclusively activates diverse cells in the local network that may be important for network function and plasticity, such as inhibitory neurons (Liu, 2004; Hensch, 2005; Mariño et al., 2005; Southwell et al., 2010) and astrocytes (Schummers et al., 2008; Perea et al., 2009).

One concern we had in designing the system was whether cellular activation in the intact network would correspond at all to electrode position. It was possible that in the intact circuit, axons would be preferentially activated and at times carry the stimulus great distances antidromically to far away cell bodies (Histed et al., 2009). However, based on the visualization of whole network responses through calcium imaging (Figures 2G, 6), and direct recording of cell bodies through intracellular patch clamp (Figure 2), we observed good correspondence between electrode position and the position of the activated neurons, with the radius of activation increasing systematically with increasing voltage, as intuitively expected. Another concern we had related to synaptic transmission, and whether it would be straightforward to find synaptic partners that could provided drive to a cell of interest. Fortunately, it was a quick and routine part of an experiment to stimulate an electrode and easily come in contact with synaptic partners; tuning the stimulus amplitude could in turn refine the activation down to a single monosynaptic partner (Figure 3C). Thirdly, we were concerned that given the unknown nature of stimulating multiple sites in fast patterns, there might be strange electrical interactions between the electrode substrate and the neuronal circuit, making stable responses difficult to obtain. However, the weighted slices remained closely apposed the substrate throughout the experiment, and action potentials (Figure 2) synaptic transmission (Figure 3) and calcium responses (Figure 6) all persisted at stable levels for hours during the patch or imaging sessions, as observed in comparable single electrode stimulation studies.

### Fast Activation Patterns Enable the Exploration of New Regimes of Multi-Neuronal Activation Relevant to Plasticity

Based on previous studies of plasticity using similar probe arrays, which found that plasticity was difficult to induce via stimulation from various electrodes (Wagenaar et al., 2006), it was unclear whether or not our patterned stimulation platform would be an effective tool for studying plasticity. Previous studies, however, attempted to induce plasticity in dissociated culture, which is known to demonstrate limited plasticity in the absence of specialized pharmacological conditions (Slutsky et al., 2004) (though see Chao et al., 2008). We thus adapted our platform to acute slices of rodent cortex (Oka et al., 1999), to create an environment more favorable for inducing plasticity (Cristo, 2001). Here we first employed canonical homosynaptic plasticity paradigms, by applying tetanic stimulation to sequentially drive potentiation at two synaptic inputs to the same cell (Supplementary Figure 3).

Using this plasticity paradigm, we successfully induced two independent plasticity events, demonstrating that plasticity could, in fact, be induced via our stimulation platform. Encouraged by these results and given the straightforward control over multiple, independently driven inputs which our platform could provide, we began to explore new plasticity paradigms, enabled by the ease of use and versatility of the stimulator's software-programmable electrodes. In the next successful paradigm that we arrived at (Figure 4), we identified two synaptic inputs, one superthreshold (“strong”) and one subthreshold (“weak”), and by stimulating the “weak” input in close temporal proximity to the “strong,” we were able to selectively potentiate the “weak” input, while maintaining efficacy of the “strong” input. These results indicate that the system can be useful for investigating parameters of coincidence detection (Frégnac et al., 1988), spike-timing dependent plasticity (STDP) (Markram, 1997; Bi and Poo, 1998; Song and Abbott, 2001) and heterosynaptic capture (Royer and Paré, 2003), as well as exciting emerging mechanisms (Harvey and Svoboda, 2007; Losonczy et al., 2008; El-Boustani et al., 2018) by which multiple synaptic pathways communicate.

In a similar exploration of activating neurons sequentially with fast temporal precision, we employed an all-electrode fast spatial pattern, resembling a “moving bar” of sequential activation moving horizontally across the cortex (Figure 5 and Supplementary Figure S6). Here we observed significant strengthening of total responsiveness, and an increase in the likelihood of neuronal spiking, following repetitions of the pattern. Such enhancement could arise as simply an extended form of STDP, or from related mechanisms of sequential feature detection that operate at the cellular or network level (Fiete et al., 2010). Fast, spatiotemporal stimulation thus allowed us to investigate plasticity between neurons and has the potential to provide insight into the mechanisms underlying it.

### High Throughput Calcium Imaging Combined With Multi-Site Stimulation Enables “Population” Network Physiology

Of particular promise is the ability to conduct “population” multi-site stimulation during simultaneous “population” imaging of calcium responses (Figure 6). This offers the promise of conducting measurements of site-to-site activity and transmission without requiring laborious patch clamp experiments; instead slices can simply be placed on the array and immediately imaged during patterned stimulation.

The “plasticity status” of a brain circuit can thus be assessed without any manual intervention, providing a mechanism for the screening of compounds designed to change brain plasticity. Also, the present work showcases plasticity using a CCD camera, but this might be readily swapped with two-photon imaging to assess the functional status of single cells at the population level. Combining this methodology with cell-type specific labels could allow one to discern the role of different cell classes during the induction or expression of plasticity.

### Population Stimulation Enables New Investigations in Circuit Plasticity, and Holds Relevance to Future, Optical Stimulation Systems

Numerous applications become immediately available once the type of patterned “network” plasticity demonstrated here becomes more commonplace. For one, synaptic physiology benefits because multiple presynaptic partners can now be driven in tandem while measurements of synaptic transmission and plasticity are obtained (Royer and Paré, 2003), providing an experimentally trivial way to activate many neurons to explore heterosynaptic interactions based on timing or circuit location. Second, our methodology suggests a more “high throughput” means for assessing transmission and plasticity, by “pinging” various network locations while assaying function with calcium imaging. In this case we focus on measurements before, during and after plasticity events driven by patterned stimulation, but it could easily be invoked for drug screening studies that measure transmission or integration between different areas over time while a compound is applied to the circuit. Network-level activation could also be coupled with the mapping of the connections that underpin the transmission and plasticity (Zolnik et al., 2016; Izquierdo-Serra et al., 2018). To our knowledge, developing this type of high throughput screening method for functional transmission remains an open opportunity. Finally, the platform may have applications down the road for the “programming” of neuronal circuits during prosthetic rehabilitation (Donoghue, 2002; Nicolelis, 2003; Jepson et al., 2014) - being able to co-activate neurons on demand or trigger them in sequences could facilitate the re-learning of tasks following injury, or the learning of new tasks.

Driving networks in fast, distributed patterns is a principle of experimentation that may 1 day be a staple of neuroscientific inquiry. Irrespective of the end-point stimulus actuator, we found that complex logic is needed to design, route, and manage the spatiotemporal stimuli and their responses. Indeed, even once we had established a hardware architecture for controlling the distributed pulses, tracking them required software design and engineering that will be requisite to future multi-site interfaces, whether they take the form of multiplexed laser beams (Nikolenko et al., 2007), multi-patch arrays (Hai et al., 2010), LED arrays (Grossman et al., 2010), or rapid lasers that can approximate simultaneous activation (Shoham et al., 2005; Wilson et al., 2012). Such brain stimulation will generally be reducible to the digital-to-analog production of signals that then control 2 parameters: stimulus position (via electrode gating, mirror position, or acousto-optic deflector (AOD) frequency) and stimulus intensity (via electrode amplitude, LED brightness, or laser pockels cell). These same parameters are used in managing the patterned activation sequences presented here.

The most obvious future improvement in our multi-site stimulation system (see also supplementary document, “System Limitations and Future Opportunities”) will be to adapt the system to one that can focus multi-cell stimulation at single cell resolution, and recent studies (Daie et al., 2021) have demonstrated the promise of this approach, which might now be extended to the cell population level to drive network plasticity. The only way to currently stimulate targets with cellular resolution is to invoke the precision of a two-photon laser (Rickgauer and Tank, 2009; Daie et al., 2021). The limitation here is that a two-photon laser cannot be in multiple places at the same time, and sufficient dwell time is required to activate a given cell, such that the laser would have to be directed in a rapid “start stop” sequence between cells, utilizing arbitrary beam paths that minimize travel distance in order to effectively be in “many places at once.” Recent work showcases how this might be approximated with AODs (Shoham et al., 2005) or spatial light modulation (Peterka et al., 2010). Progress in arbitrary beam paths for two-photon imaging (Lillis et al., 2008; Wilson et al., 2013) might also be re-purposed for two-photon stimulation.

### On Automation in Neurophysiology

Computer-controlled, closed-loop awareness of the full spectrum of stimuli and responses, and their registration in time and space, allow the guided delivery and refinement of complex stimulation (See also supplementary document, “Steps Toward Automated Experiments and Analysis”). In the experiments presented here, the stimulation pattern could be downloaded to the circuit in advance, and dispatched in coordination with acquisition and real-time analysis to achieve high-level awareness of the circuit's evolving plasticity status. In the future, that status could be converted to an objective function that guides the delivery of the next iteration of stimulation, to either guide/sculpt the circuit toward an outcome, or search for optimized stimulation patterns that optimize toward an outcome.

## Conclusion

At the heart of this work is the notion of “matrix stimulation” in which multiple sites of a neuronal network are driven in parallel or asynchronously at the discretion of the experimenter. We wished to develop a robust methodology for assessing activation, transmission and plasticity between neurons while engaging multiple cells in fast patterns, and employing either electrical or optical readouts of activity. Our belief is that even as technology advances, and whether future experiments consist of hundreds of lasers *in vitro* or arrays of “optrodes” *in vivo*, they will still be controlled by electrical pulses emanating from many channels that are orchestrated in space and time and linked up to the responses of populations of neurons; our work offers a step in this direction.

## Data Availability Statement

The raw data supporting the conclusions of this article will be made available by the authors, without undue reservation.

## Ethics Statement

The animal study was reviewed and approved by MIT Committee on Animal Care.

## Author Contributions

NW and MS conceived the system and experiment design, refined the manuscript, and experiments were carried out by NW, FW, SY, and NC. AD, BS, and NW conceived the electronics. AD designed and built the analog circuitry. BS designed and built the digital circuitry. SS refined the circuitry and helped refine the system and experiments. NC developed the concurrent electrical and optical approach. NW and FW prepared the text and figures. All authors contributed to the experiments and system design.

## Funding

This work was supported by NIH grants EY007023, MH085802, and MH126351 to MS, and postdoctoral fellowship EY017500 to NW.

## Conflict of Interest

NW is now employed at Nara Logics, Inc. The remaining authors declare that the research was conducted in the absence of any commercial or financial relationships that could be construed as a potential conflict of interest.

## Publisher's Note

All claims expressed in this article are solely those of the authors and do not necessarily represent those of their affiliated organizations, or those of the publisher, the editors and the reviewers. Any product that may be evaluated in this article, or claim that may be made by its manufacturer, is not guaranteed or endorsed by the publisher.
