# Supplementary material for: A Platform for Spatiotemporal “Matrix” Stimulation in Brain Networks Reveals Novel Forms of Circuit Plasticity
Source: Front Neural Circuits. 2022 Jan 5;15:792228. doi: 10.3389/fncir.2021.792228 (PMC8766665; doi:10.3389/fncir.2021.792228)
Supplement: Supplementary file 2 [file Data_Sheet_2.PDF]

Supplementary Figure 1: Mechanism of spatiotemporal stimulation across 64 channels.

(1a) Working from any computer with a USB port, the experimenter defines stimulation matrices in a matrix-capable programming language like MATLAB or Python. The stimulation matrix consists of an arbitrary number of stimulation events across 3 columns, each denoting 1) the electrode number, 2) the time in ms that the stimulus pulse should arrive, and 3) the strength of the stimulus pulse at that electrode. An arbitrary number of rows can then be used to denote simultaneous or asynchronous stimulation events within the pattern. **(1b)** This matrix is sent via a Matlab command over USB to a commercial FPGA (Xilinx Spartan-III, Opal Kelly), using a driver included with that FPGA. The pattern is now stored in the FPGA's memory for real-time triggering. The specific hardware architecture for our stimulator can be downloaded or "programmed" onto any such purchased FPGA. (**1c)** Upon receiving a "go" command from the computer over USB, the programmed FPGA generates the voltage commands for the pattern in real-time, which are sent over an 8-bit data bus to a digital-analog converter (DAC). The DAC translates these voltage commands into actual voltages which are sent out towards the electrodes. (**1d)** Only one electrode at a time will sample each voltage level, and that electrode is selected by a rapidly cycling 6-bit address sequence (more bits can be easily added to address more electrodes). The timing of the cycling address sequences is synchronized with the voltage commands, so that each electrode is activated at the exact moment that its corresponding voltage level is produced by the DAC. (**1e)** Each electrode is then equipped with a "sample and hold" module, so that upon activation by its address sequence, the electrode "samples" the synchronized voltage level from the DAC, and then "holds" it in preparation for coordinated release with the other electrodes. The speed of loading all electrodes with new voltage levels and triggering "release" depends only on the interval of the FPGA clock, which can be at MHz speeds. In our stimulator we set the clock to update the voltage levels across 64 electrodes with microsecond precision. (**1f)** As the multiple electrodes are coordinately fluxed with dynamic voltage levels over time, the experience at any connected electrode probe is one of real-time distributed spatiotemporal stimulation, controlled from a standard computer environment.

Supplementary Figure 2: Versatility of spatiotemporal activation patterns and functional interface

(2a) The system can in principle be used at multiple scales depending on the effector, from electrodes distributed throughout an animal, to a microclustering about a dendritic arborization - we connected ours to planar arrays of electrodes (200 µm spacing) interfaced to acute slices of mouse visual cortex (**2a, left**). Scale bar: 200 µm. Networks of cells in our slices could be stably imaged and healthily maintained in ACSF during stimulation for up to 12 hours (**2a, right**). Scale bar: 30 µm. To test the versatility of the system, we also functionally stimulated dissociated neuronal cultures that were grown on the probes (**2b**), as well as slices through electrode arrays of finer scales (10 µm spacing, not shown). Scale bar: 100 µm. Stimulation channels could be driven with arbitrary amplitude and timing, independently or simultaneously (**2c, left**) and measuring the output of the stimulator verified specific routing (**2c, right**). As an example of the spatiotemporal nature through which stimulation could dynamically drive an intact network, we connected the stimulator to a cortical slice bulk-loaded with calcium indicator to report activation, and applied a "moving bar" of electrode activation (**2d**) moving from left to right across each column of electrodes at 100 ms intervals. Scale bar: 200 µm. Measuring at regions of interest (ROIs) in each column during the activation pattern (second row of circles), we were able to detect distinct responses at different points in the network as the pattern reached that location, suggesting that the electricity is precisely routed in space and time (**2e**, see also Figs. 2, 6). Scale bars: 400 ms, 20% $\Delta F/F$.

Patterns might in principle also be delivered to circuits at single locations (**2f, left**), alternating patterns of active and inactive "grids" of arbitrary amplitude, to explore spatial encoding by distributed networks (**2f, right**), or even spatially distributed activation intensities (in frequency in amplitude) that approximate a "natural scenes" visual stimulus (**right**). While more straightforward network activation experiments immediately arise, the stimulator should make possible an experimenter's activation pattern of choice, with precise timing and minimal coding, to selectively engage different sites in a network. Software control from a personal computer interface (in conjunction with dedicated hardware) means that patterns can be rapidly adjusted or swapped during the experiment, or influenced by machine learning algorithms or other software systems to optimize stimuli for the network activation or plasticity phenomenon of interest.

The data shown above was taken from a slice that had already been stimulated with various patterns for over 10 hours, retaining its dye and functional connection throughout this period.

The system thus provides a robust interface for repeatable activation for a lengthy duration, which could be applicable to the testing of drug compounds or extended plasticity assays.

Supplementary Figure 3: Induction of multi-site synaptic plasticity

As a first test to see whether our system could induce plasticity, and particularly whether sequential stimulation could elicit plasticity at multiple synaptic pathways, we sought to use a well established homosynaptic plasticity paradigm to potentiate transmission at one synaptic input, measure a change, and then potentiate transmission at a second input to the same cell. Tetanic stimulation is a reliable way to drive potentiation at synaptic pathways in cortex. In these experiments, an acute slice was again interfaced to the multi-electrode probe (**3a,** scale bars: 400 μm). A neuron was patched, and presynaptic stimulation locations were identified that produced reliable excitatory postsynaptic currents in the patched cell of interest (**3b**) Scale bars: 100 ms, 120 pA. One input location (Input A, 3a) was then selected at which to deliver a tetanic stimulation pattern, in order to potentiate its relationship with the patched cell, which was also released from voltage clamp during the tetanus. After a reliable number of pulses had been delivered to establish a general baseline strength (at 0.2 Hz, **3c**) for the input, tetanus was delivered, and the input strength was subsequently resampled to evaluate whether the strength had changed (**3c**). Comparison of averaged EPSC waveforms from before and after tetanus revealed an obvious change in EPSC amplitude (**3e**, left traces, scale bars: 50 ms, 50 pA), and a statistical increase in peak amplitude (**3f**), indicating that the tetanus had been effective at potentiating the first input of interest. We then immediately moved to a second input location (Input B, 3a) and delivered pulses to assess its general baseline strength (again at 0.2 Hz, **3d**), verifying that Input B remained at a similar baseline level to that observed prior to the tetanus of Input A in our initial mapping. Once this baseline was established, a tetanus was applied now to Input B while the patched neuron was released from voltage clamp. Resampling the strength of Input B following tetanus to this second site revealed that it too had now been potentiated (**3d**), resulting in an obvious increase in amplitude when comparing average traces before and after tetanus (**3e**, right traces, scale bars: 50 ms, 50 pA) and when quantifying peak amplitude (**3f,** N=24 events post vs. 7 pre for Input A, 41 events post vs. 21 pre for Input B). Thus, multiple inputs could be sequentially potentiated by delivering step-wise temporal programming to distinct input sites converging on a given cell of interest. Having straightforward control over

multiple inputs that can be driven independently, and that exhibit independent plasticity statuses, lays the groundwork for more interesting interactions between inputs driven in more coordinated time patterns.

Supplementary Figure 4: Production of arbitrary time-varying signals.

An advantage of facile computer-control over waveform design is that it is possible to generate any time-varying function (at least within the temporal resolution of the system, described in the supplementary document, "Analysis of Temporal Precision", and Figure 2).

To demonstrate smooth waveform production in shapes beyond the square pulses utilized for neuronal activation, we generated a sinuosoidal waveform and captured the resultant voltage signals coming out of the stimulator. **(4a)** A sinuosoidal stimulus oscillating between +/-1.5 V, output at one sample-and-hold channel of the stimulator. **(4b)** A different sinuosoidal stimulus, now oscillating at a different voltage level with an offset.

As described in Materials and Methods, arbitrary stimulus waveforms can be defined using any software of choice that is capable of capturing numerical vectors or arrays, and this could be facilitated via custom graphical user interfaces or stimulation studio software applicable to the task at hand.

Supplementary Figure 5: Automated detection and analysis of electrophysiological events.

(5a) A cortical slice is readily interfaced with a planar multi-electrode array, by placing it on top of the array with a weighted net to facilitate contact. Perfusion tubes, weighted net, intracellular patch clamp pipette, and imaging lens can all be made to fit in the electrode array chamber after some careful positioning. **(5b)** Intracellular patch clamp can be readily obtained using standard methods, with clearance of the electrode array chamber and upright objective lens by the recording pipette possible with 10-40x lenses. Intracellular patch clamp could be readily achieved and was not disrupted or lost by the patterned electrical stimulation. Recordings with robust spontaneous activity **(5c)** could often be maintained for hours which facilitated some of the long-term plasticity explorations as described. Scale bars: 200 ms, 40 mV. **(5d)** Routinely obtained patch recordings in this configuration afforded collection of measurements from a

variety of cells on each slice, and the capture of clean synaptic events that could be passed through automated analysis as data was acquired. 1 second sweeps were recorded, and automated detection applied (see Material and Methods). **(5e)** This resulted in the collection of large volumes of synaptic events with distributions of attributes that could be compared before and after patterned stimulation-driven plasticity events. **(5f)** Other attributes can be automatically tracked for change in response to plasticity events, such as amplitude, frequency, and rise and decay time. Electrophysiological readouts can be swapped for automated imaging-based ones, which in conjunction with the patterned stimulation provide a more high-throughput means of patterned stimulation and functional readout.

Supplementary Figure 6: Extended time course of response changes during patterned stimulation.

(6a) Inset: A layer 5 pyramidal cell was patched and filled with Oregon Green for concurrent imaging while intracellular recording was held for hours during the delivery of patterned stimulation. Scale bar: 25 μm. **Time course:** First 5 time points – a "baseline" stimulation protocol was carried out, involving sampling responses across the multi-electrode array in a grid pattern one by one. Spikes were not observed in response to these test pulses. **i.** A "moving bar" stimulation paradigm was applied, in which a vertical bar was "moved" horizontally across the electrode array, by stimulating one column of electrodes after another in fast succession (50 ms delay between columns). Columns were activated in this manner in the order {1, 2, 3, 4, 5, 6, 7, 8}, and this stimulus was repeated 100x (100 sweeps). At each dot, this moving bar stimulation pattern was repeated, while sampling spikes in response to each of the stimuli sweeps. **ii.** After a 20 minute "rest", the stimulation pattern was repeated, and now more spikes were observed per sweep. As a control during this period, we also did a "random" sampling of individual electrodes in a random order and checked for spikes. **iii.** Waiting still longer after the initial moving bar and repeating the stimulation protocol, we now observed a dramatic response to the moving bar. **iv.** To see if spiking could be abolished, we attempted to "erase" the patterned response, by applying bars in a random order of {2, 7, 4, 1, 6, 3, 8, 5} to contrast with the original trained pattern. We saw that this resulted in a reduced response level. **v.** To see if the originally trained pattern was retained after these changes, we repeated the original moving bar stimulus, and observed an elevated response again. **(6b)** Time course of the response rates during the applied stimulation

itself, where the # of spikes was tracked for each sweep, to compare the variance across sweeps. The original moving bar produced consistent responses when applied many times, corresponding to time points (i) and (ii) in (A). **(6c)** An “erase” or random pattern corresponded to a gradual rundown of spike rate over the course of the stimulation. **(6d)** Sample action potential responses during stimulation at each of the various points in the time course. Scale bars: 100 ms, 30 mV. As shown on the inset in (iv), applying an IV test pulse to the interior of the cell from the patch pipette still resulted in robust spiking even after hours of the patterned stimulation, indicating the capacity to record and measure changes to cells in the circuit over hours of spatiotemporal pattern delivery.
