# Supplementary material for: A Platform for Spatiotemporal “Matrix” Stimulation in Brain Networks Reveals Novel Forms of Circuit Plasticity
Source: Front Neural Circuits. 2022 Jan 5;15:792228. doi: 10.3389/fncir.2021.792228 (PMC8766665; doi:10.3389/fncir.2021.792228)
Supplement: Supplementary file 3 [file Data_Sheet_3.PDF]

Temporal precision across many channels during patterned stimulation

The goal of the design presented here is to provide arbitrary waveform control from a typical PC across 64 independent analog channels, with sub-millisecond temporal resolution, and predictable timing coordination across channels or sweeps. What follows here is an analysis of the bottlenecks and opportunities encountered towards achieving this crucial degree of system fidelity.

Temporal precision – computer

The key to this stage is to fully remove the computer and USB from the timing control loop. Most lab workstations continue to be personal computers running software like Windows or Mac OS, which make no guarantees about temporal fidelity and must balance multiple applications and system resources that are being prioritized.

Temporal precision - USB

Similarly, while USB exceeds our requirements for transmission speed, we also have the burden of minimizing the latency in data transfer. Unfortunately, this is an area of weakness for USB, which excels at transferring large chunks of data but is considerable slower at sending periodic small chunks of data. We developed a test program to validate the onboard waveform storage, and found that we were able to transfer data to the FPGA at anywhere between **1 Mbit/second and 10 Mbit/second**. These values are well below the theoretical limit for USB. Part of the performance hit results from additional overhead introduced by our use of the Opal Kelly USB module. Despite this, we have more than adequate bandwidth to get our stimulation patterns across, but not with the certainty of arrival time that precise stimulation delivery mandates.

The goal at this stage is therefore to design the orchestration patterns on the PC via the convenience of software, but then deliver those patterns safely to high-performance onboard memory where they can interact with digital triggers for true temporal precision. This is what we did in this design (see Materials and Methods) and therefore the computer and USB bus have no bearing on the operational temporal precision.

Temporal precision – digital control circuitry

The digital logic that handles the control signals can reach clock speeds of hundreds of megahertz and is not a bottleneck in the system. Furthermore, the use of RAM in the hardware to cache output waveforms reduces the amount of data going through the USB link such that in practice, the entire operational control sequence can be downloaded off the computer and then run with high precision over hours (since most of these waveforms are the same stimulation patterns or test pings run in a loop).

Temporal precision – analog dispatch circuitry

With the digital logic orders of magnitude faster than needed, and the operating system cut out of the equation, the principal remaining locus for time delays was the analog circuitry. The integrated circuits used in this design were therefore chosen in part for their speed. The TLC7628 DAC has a propagation delay of 180ns and the SMP18 sample and hold has a propagation delay of 3.5us, making the total propagation delay of the analog circuitry (the delay between when the databus bits are updated and when a new voltage appears at the output of a sample and hold channel) approximately **3.68us**. Therefore each frame within a time window, as defined by the controller, must be at least 3.68us, such that the data bits designated for one channel are transformed into an analog voltage before the next channel is dealt with. To ensure no errors, we set this parameter to **5us** within the controller's timing module, to also account for any extra small delays induced by op-amps, etc. Given this parameter, the voltage across all 64 channels can be at a speed of **5us*64 = 0.32ms**, thus meeting our goal for sub-millisecond control across all channels.

Temporal precision within and across channels

Channels are thus updated at specific microsecond offsets that amount to a grand total of a fraction of a millisecond, and these offsets are known quantities that can also be pre-compensated in the digital circuitry design, alongside a regular clock cycle, to exercise a predictable impact on the looped pattern, where exercising the same control pattern in a loop will lead to the same precise and repeatable phase in time across channels.

To confirm the temporal precision or lack of “jitter” across channels as current was dispatched across multiple channels with a recurring master clock cycle, two different waveforms, of identical time signature but completely different baseline amplitudes, were dispatched to distinct channels across the array:

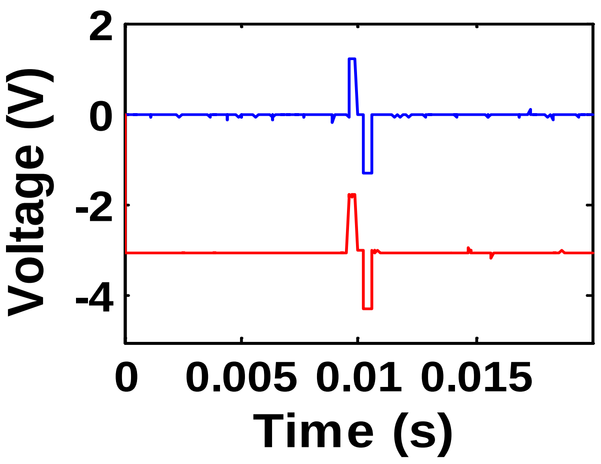

Sub-millisecond temporal precision across channels, with high-fidelity time synchronization.

We therefore observe the following in terms of the system’s timing precision:

- Pulse timing and waveform shape can be precisely controlled to manifest as designed at the PC.
- All channels can be updated with sub-millisecond timing resolution.
- Channels are well synchronized with one another, with phase fidelity that is precisely maintained across sweeps and between channels.
