# Supplementary material for: A Platform for Spatiotemporal “Matrix” Stimulation in Brain Networks Reveals Novel Forms of Circuit Plasticity
Source: Front Neural Circuits. 2022 Jan 5;15:792228. doi: 10.3389/fncir.2021.792228 (PMC8766665; doi:10.3389/fncir.2021.792228)
Supplement: Supplementary file 4 [file Data_Sheet_4.PDF]

Open hardware and software components

Computer

- Software – MATLAB, Python, etc.
- Operating System – Microsoft Windows
- USB cable

Data acquisition alongside stimulation

- Data acquisition cards – National Instruments PCI-6110 (NI.com)
- Breakout boxes – National Instruments BNC-2110 (NI.com)

Digital Controller

- Hardware – Opal Kelly XEM3001 / Xilinx Spartan-3 (Opal Kelly)
- Software – Verilog HDL

Key Analog Components

- Digital to analog converter – Texas Instruments TLC7628 Dual 8-bit, 0.1 µs (Mouser)
- 8-channel sample and holds – Analog Devices SMP18 (Analog Devices)
- 3-to-8 line decoder/multiplexer – Texas Instruments CD74HC237E (Digikey)
- Operational amplifiers – Analog Devices OP495 (Analog Devices)
- Power supply – Power-One MAP55-4000 (Digikey)

Integration

- Circuit board fabrication – manufacturing service (ExpressPCB.com)

Interface connectors

- MEA cable adaptation – National Instruments SCB-68 Shielded 68-pin I/O block (NI.com)
- MEA connector cable – National Instruments SCH68-68, 68-pin SCSI cable (NI.com)

Brain interface

- Electrode array housing – MultiChannel Systems MEA-60 (MCS)
- Multi-electrode array – MultiChannel Systems MEA-60 (MCS)
