# Supplementary material for: A Platform for Spatiotemporal “Matrix” Stimulation in Brain Networks Reveals Novel Forms of Circuit Plasticity
Source: Front Neural Circuits. 2022 Jan 5;15:792228. doi: 10.3389/fncir.2021.792228 (PMC8766665; doi:10.3389/fncir.2021.792228)
Supplement: Supplementary file 5 [file Data_Sheet_5.PDF]

Steps Towards Automated Experiments and Analysis

We employed several automation patterns to support end-to-end computer control of the details of experiment and analysis execution, to allow the experimenter to focus on the high-level interplay with the network being explored. Here we try to share some of these, in support of investigations where observed responses can be readily collated with the precipitating stimulation patterns without much effort, to produce a picture where the experimenter can then quickly define the subsequent stimulation patterns and maintain cadence with the preparation.

I. Setup - Standardized data acquisition and analysis

Coordination between subsystems is clearly simplified when they share a common data acquisition control plane. For this, all acquisition and analysis has been consolidated into the same MATLAB software environment (Network Prism, Sur Lab):

- Electrophysiological data acquisition
- Integrated electrophysiology amplifier control
- Patterned stimulation control
- Imaging system control
- Analysis as acquired sweeps arrive

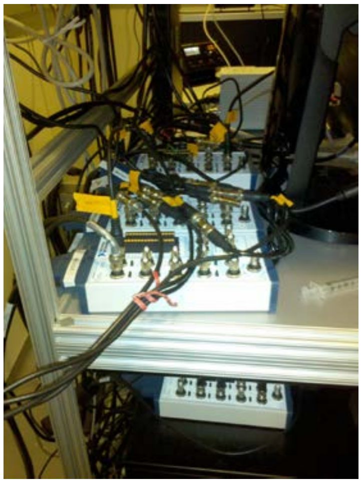

The key software drivers and hardware used were:

- **Computer:** Dell desktops, ideally with a large chassis for the cards
- **Operating System:** Microsoft Windows
- **Underlying software:** MATLAB, utilizing the data acquisition toolbox
- **Data acquisition cards:** National instruments PCI-6110 and similar
- **Breakout boxes:** National Instruments PCI-6229

This allowed custom analysis routines to take place within the acquisition software, on the data as they came into memory.

Similarly, there was joint awareness in the active data structures of designed stimulation patterns and patch clamp recording sweeps, and their timing registration, since the same software orchestrated both, and this facilitated the attribution of responses to the stimuli that caused them.

For example:

- The spatial positions of electrodes and circuit pin mappings were defined in advance, and could be intersected with data on the electrode array used that day to immediately know the absolute position of stimuli
- These could in turn be intersected with the telegraphed imaging commands to support image registration and ROI computation
- In other words, the time and space to focus on in the analysis was supported by having all of the primary facts arranged in order to produce the experiment.
- This also allowed for higher-level deductions, where knowledge of the pattern structure across time (pins or sequence comprising a coherent "moving bar") could be linked to the analysis patterns (was there a sequence detected at the right times and places in response to the sequential activation), using analysis mappings to make it easier to keep track of the different changing dimensions, rather than trying to recapitulate them across systems

Finally, the data acquisition hardware and triggering were standardized, to support cross-triggering and real-time coordination.

II. Progressive enhancement of data

We would start one data file and then build it up, adding layers to it as more information became available. For example:

- We would start with an empty recording with the full system configuration captured, preparing to layer in recording sweeps as they arrived.
- Automatically detected events were then layered into the same data structure (in a different namespace) alongside the raw data.
- A third layer could then be computed, in which aggregate statistics were quickly automatically computed and reported.
- We tried to gather as much information as we could on the state of each subsystem, but also provide higher level analytics that allowed quick-glance reporting on the physiological state of the experiment, particularly around plasticity that might be arising (changes in functional properties at the aggregate level).

III. Typical workflow

First, the experimenter designs a given orchestration pattern of distributed stimulation to deliver over time to the circuit. Given the high-level matrix language and further method parameterizations (spatial pattern components, "speed", timing settings, etc.), these can be generated quite quickly as they occur to the experimenter

Then, they can hit "go" and the following would proceed automatically:

1) the stimulation pattern will be downloaded to the circuit, for high fidelity reproduction
2) a set of digital triggers will initiate both the recording and stimulation systems, acquiring data in a loop while presenting the complex stimuli, and
3) automatic event detection, quantitation and analysis occur as data is coming in, with the potential to notify when certain conditions (% change, etc.) are met in the circuit.

We would typically cycle through a few periods of baseline in this manner to get a sense of the circuit's state. We would then trigger the stimulation patterns designed to drive plasticity (these patterns were usually coded in advance of the experiment but could also be improvised on the fly due to the facile interface. Finally, we would capture more cycles while monitoring analysis results to see if plasticity was setting in.

IV. Degree of actual end-to-end automation

For electrophysiology, the workflow was pretty one-touch from triggering through analysis.

For imaging, while analysis of the full-field image sets was fully automated, they necessarily take place right after the acquisition / stimulation has concluded, and are initiated manually, because the processing time takes longer per frame than the speed of acquisition. But increasingly compute power should eventually make this real-time as well.

Still underexplored areas with interesting potential:

The potential of analysis results to loop back and directly modulate the future stimulation patterns presented are an interesting future possibility – this is where things get especially interesting, and you could conduct optimization routines towards:

- Closed-loop search algorithms, which
- Guide the circuit towards an outcome, or
- Search for patterns that optimize towards an outcome.

V. Further analysis detail

Here we provide detailed analysis procedures that could be run autonomously in support of the closed-loop analyses. See also Supplementary Figure 5 for an illustration of some of these approaches.

A) Automated response detection – electrophysiology – action potentials

For every 1 second sweep, the baseline was calculated by taking the median value for the sweep, and a detection threshold established at 7 standard deviations from the baseline. At the first deflection that crossed this threshold, we cut a window from the first deflection point forward by a standard width

(usually 1 ms) and searched forward either to the maximum of the window width or the first point where it returned to baseline, whichever came first. The maximum point detected in this window was considered the peak, which we tagged as the spike time.

B) Automated response detection – electrophysiology – synaptic currents

Here we had the advantage of knowing the precise time in the sweep at which a stimulus was delivered, to know where to search for the response, if any. We would compute the baseline by averaging data from the 2 ms prior to the potential evoked event. We would then step forward, while averaging a 1 ms window on either side of a search cursor. The rise time was considered started at the first point where deflection from baseline exceeded an average of a threshold value across the window, usually of 1-2 pA. The decay time was considered the point where the search window returned to baseline. The event peak was taken as the position of the search window that when centered produced the maximum deflection across the points averaged, and the center of the search window at that point was designated the event time. For completeness, the stimulus transient was also captured and tagged.

C) Automated response detection – imaging / optical events

Images were continuously acquired at 2.5x, 10-20 Hz, using 4x4 binning. Regions of interest (ROIs) were generated by our software automatically, by juxtaposing a 2D map of the stimulated electrodes onto the imaging plane, and expanding a circle around each electrode point so that radii between circles were maximal but non-overlapping. The mean fluorescence for each ROI was tracked from frame to frame, resulting in a time-varying intensity signal for each ROI. Correction algorithms were applied to subtract a global fluorescence depletion (bleaching) slope over the course of the experiment via detrending, and global macro events that impacted all ROIs equivalently (lamp flicker noise), often by subtracting the scaled full-frame signal from the ROI's signal, to provide a flat baseline sweep without macro disturbances to then zero in on deflections that were due to functional events specific to the ROI. dF/F responses were quantified for each stimulus by analyzing the time-varying signal within each ROI, and computing the change in deflection from the baseline, where the baseline was calculated as the median value over the 1 s sweep corresponding to the stimulus, as above for the synaptic current analysis. Also as with that analysis, the peak was detected by searching forward from the stimulus event with a moving average window, and searching forward for deflections that exceeded a threshold, usually 2-3% dF/F.

VI. Further potential for extending the automation to spikes in cells across the network:

The following additions could allow for a "network view" of spiking across distributed cells, allowing population cellular responses in conjunction with population stimulation. We have used these approaches fruitfully with higher-resolution (two-photon fast-scanning) imaging systems, and it would be interesting to combine them with the population stimulation.

A) Cell segmentation for cellular-based responses. Our software also implements a fast cell segmentation method developed in our lab and optimized for neurons and astrocytes. The basic premise is to apply a "watershed filter" that raises the "water level" on the background until only the "peak plateaus" (cells) of pre-specified radii are "above water" as determined by comparing a noisy 2-dimensional Gaussian and filling in the "convex hull" areas of a common intensity level. In practice in our software, the user has control over two parameters that let them specify the typical "cell radius" for the software to expect (which uses the user-specified "zoom" and "lens magnification" to determine the actual size of what it is detecting), and then adjust the "threshold" which corresponds to the water level to subtract the background and isolate the cells.

B) Deconvolution of optical signal to spikes. We also worked towards implementing a previously published deconvolution algorithm that we found especially helpful (Grewe et al., 2010). Here, a "canonical" dF/F waveform corresponding to one action potential is specified by the user, by adjusting several parameters corresponding to the dF/F amplitude, time courses, etc. The parameters can be adjusted and "run" on the waveform to assess quality of fit. The canonical dF/F waveform ("template") is then applied to the actual dF/F trace (which can be optionally low pass filtered to reduce noise) using the following steps: First, the canonical waveform is marched along the actual trace until it encounters a dF/F "bump" that crosses a simple threshold. The template is then positioned at this point, and "peeled off" or subtracted from the actual waveform. This reduces the actual waveform by the amount of the template at that location. A second template is then marched along the actual trace again, starting at the beginning as before, and this time may surpass the previous point and encounter a new point that is above threshold, where it also is "peeled" or subtracted from the actual waveform. This process continues until the actual waveform has been reduced by the set of peels and there are no further points above threshold. The locations of the peeling events, where the templates were positioned, denote the location of the likely action potentials (minus a small temporal offset to account for the delay between AP and calcium response).

Using the above additions with a suitably high-resolution imaging system, we could via optical signals reduce the responses from patterned stimulation to spikes detected in cells across the network.
