# Supplementary material for: A Platform for Spatiotemporal “Matrix” Stimulation in Brain Networks Reveals Novel Forms of Circuit Plasticity
Source: Front Neural Circuits. 2022 Jan 5;15:792228. doi: 10.3389/fncir.2021.792228 (PMC8766665; doi:10.3389/fncir.2021.792228)
Supplement: Supplementary file 6 [file Data_Sheet_6.PDF]

Limitations, Tricks, and Cautions to the Experimenter

Modern operating systems still have insufficient temporal fidelity to drive experiments

Despite recent improvements in operating systems and compute power, and the high temporal bandwidth of the improving USB interface, the operating system on standard computers remains fully unreliable at dispatching commands with the temporal precision required to interface with neural circuitry. It is therefore imperative to get the commands away out of computer memory and into onboard digital circuitry that can properly organize and trigger the waveforms in advance of the analog circuitry. This extra step was needed to actualize our system and will likely be needed for the foreseeable future.

On-board memory limitations

As the number of independent channels grows, and the desire for high temporal precision increases, a larger memory is needed to represent interesting patterns. We pushed the limits of our system by storing the waveforms as reusable fragments that could be dispatched from memory according to high-level commands. For example, a long duration 0 V signal could be created by replicating a single shorter waveform many times over, and channels with similar stimulus pulses at different times could share waveform fragments in memory. High-level sequence cues can then be provided to reconstruct and trigger the applicable waveforms, to the applicable channels, at the applicable times. This was a hack that allowed us to deliver some of the more complex patterns despite the onboard memory available in our digital circuitry – leveraging larger in-memory caches could allow for richer waveforms across more channels, beyond those employed here.

Scheduling capacity

Currently our system relies on the generation of continuous waveforms, which are expensive to store in memory when the interesting stimuli are sparsely distributed in time. One workaround is to reserve a memory block with all zeros and use it to represent delays between stimuli. A more powerful design would involve creating a more robust queue that can handle the addition of an ”offset” which would simply be a 16 bit value to delay or offset samples by a specified length of time.

Single channel sample and holds

This stage was not included in the final design because of the complexity, but it could be reintroduced if true synchrony of stimuli reaching the MEA was desired. The second stage of sample and hold would function as follows: Once all of the stimuli have been held at the channels to be active during a given clock cycle (at the outputs of the 8-channel S&Hs), the global enable signal is asserted briefly. When the global enable signal is asserted, each single-channel S&H will in synchrony stream the input signal directly to its output. Once the global enable signal is de-asserted, the single channel S&Hs will hold their outputs until the next set of signals is ready to be sent to the MEA.

Better signal conditioning

While we did convert the circuit to an integrated PCB, and added a few signal conditioning components, more could be done to further filter or condition the circle to minimize noise from switches and the ambient environment. For applications where the precision of stimulation amplitude is paramount,

additional circuitry could be added in front of, and in advance of, the sample and holds, in order to further smooth out fine modulations in amplitude.
