# Supplementary material for: A Platform for Spatiotemporal “Matrix” Stimulation in Brain Networks Reveals Novel Forms of Circuit Plasticity
Source: Front Neural Circuits. 2022 Jan 5;15:792228. doi: 10.3389/fncir.2021.792228 (PMC8766665; doi:10.3389/fncir.2021.792228)

Technical System Detail

Here we have captured various implementation details, to the extent that it inspires and facilitates further designs to systems that can orchestrate stimulation patterns across many channels delivered to brain.

The approach described here is intended to be easy to use once implemented, so that researchers only need worry about the stimuli they want to generate at the MEA interface and not the circuitry needed to generate those signals. Once implemented, we found the design can work without issue for years.

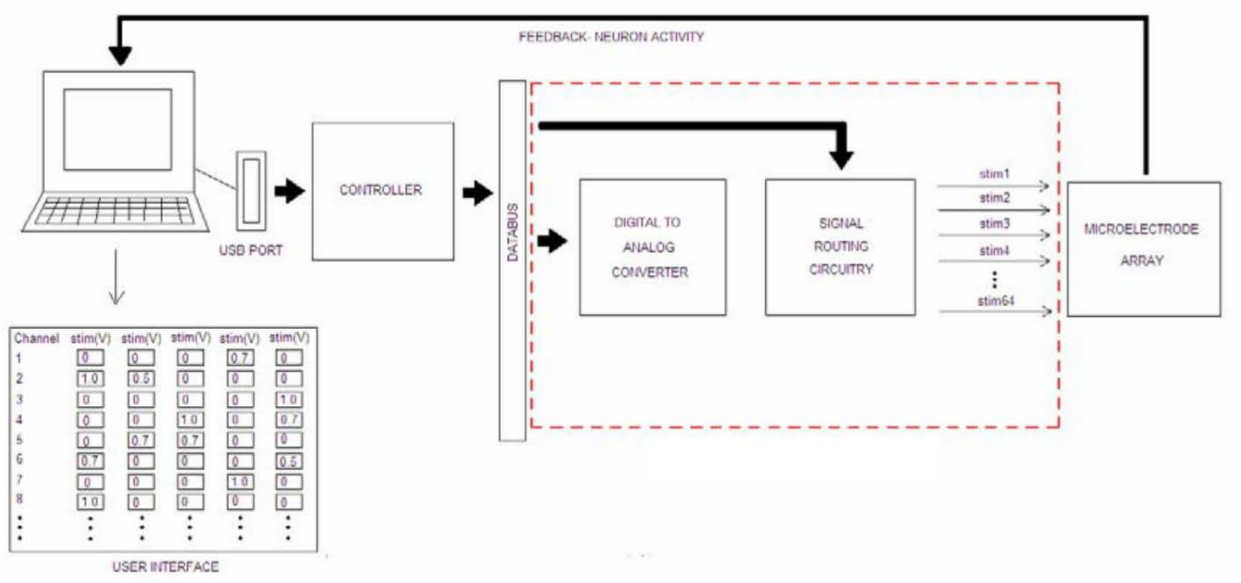

Overall workflow:

An experimenter uses a computer, connected via USB port to a digital controller. The experimenter can store up to 64 waveforms (minutes long each) in the controller's external memory, and then specify a series of those waveforms to appear at any number of 64 eventual output channels. The controller provides routing signals to a data bus, which drive a digital analog converter (DAC) and provide mapping signals to signal routing circuitry, which then distribute the analog output of the DAC across the many channels, which use "sample and holds" to sample the output at specific moments and hold that voltage level until the next sample arrives. In this way, a scalable number of channels can be controlled via patterns designed and sent from standard software in a personal computer.

User Interface:

We selected MATLAB (MathWorks, Inc.) because it is ubiquitous in academic environments and is a very powerful data analysis tool. A Universal Serial Bus (USB) link provides the connection between the computer and the controller. We selected the USB protocol to link MATLAB and the FPGA because, in addition to being available on almost all modern PC's, the protocol supports very high transmission rates.

Controller:

The controller hardware is a digital system implemented using the Verilog hardware description language. We use Opal Kelly's XEM3001v2 prototyping board which integrates a Spartan–3 FPGA and USB controller chip.

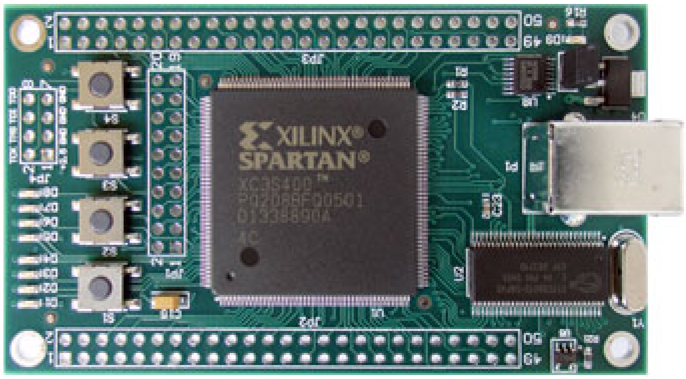

One of our primary objectives is to make this device as easy to program as possible. Opal Kelly provides a dynamically linked library (DLL) for developing applications capable of communicating with the Spartan–3 FPGA via USB.

Time-division Multiplexing:

A fundamental problem with systems which produce multiple simultaneous signals is that as the number of channels grow, the number of wires and output pins on device components quickly grow out of control. For our system, with each digital signal channel requiring eight data bits and one control bit, we would require 576 output pins on our controller for a parallel design. We use a time–division multiplexing architecture to limit the output of the controller to one 16–bit signal.

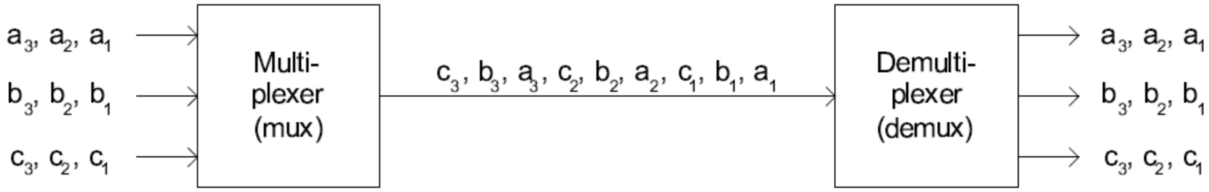

Time-division multiplexing is a method of interleaving multiple signal streams into one very fast signal stream. This has the added advantage of allowing us to easily scale the number of channels the system can support without making significant physical changes to the control signal (doubling the number of supported channels would only requiring the addition of one more bit to the data bus).

Digital logic:

The below figure shows an overview of the digital logic applied to the FPGA. We implemented this using our FPGA in conjunction with Verilog HDL:

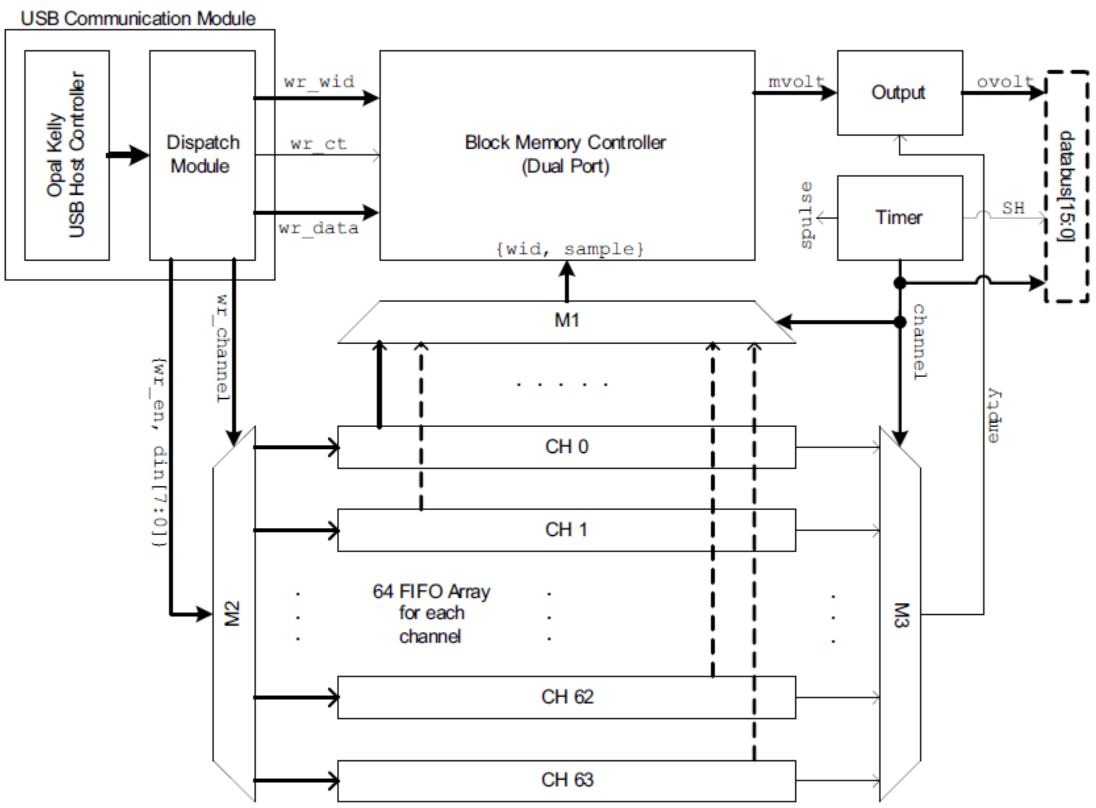

FrontPanel (Opal Kelly) provides a Verilog USB Host Controller implementation that can be used to communicate with a computer with an appropriate FrontPanel DLL. The dispatch module routes commands from the computer to the memory (to load the waveforms onto the circuit) and to the waveform queue/FIFO (to execute the waveforms). When a stimulation command is received, the waveform ID is put into the appropriate FIFO controller. Each FIFO controller has a sample counter that increments on the rising edge of the spulse signal, keeping track of the sample number. When the counter reaches the maximum value (255), the waveform ID at the head of the FIFO is discarded and the next waveform in the FIFO is processed.

The Timer module manages the channel FIFO logic. When spulse is high, it indicates that it is time to advance the sample counter by one. All non-empty channel FIFOs increment their channel counters on the rising edge of spulse. The frequency that the Timer module operates at is set by two parameters in the Verilog code. PULSE DT determines how many clock cycles each channel has available to assert its output. SH DELAY determines the amount of delay before the sample and hold signal is held high.

Sending a stimulation pattern to the digital controller:

The communication protocol layered on top of USB is abstracted from us by the Opal Kelly USB controller, or host interface. In the Verilog implementation, our module interacts with endpoints, which are data transmission/reception structures. We define two primary endpoints, one intended for transmitting waveform data and one intended for transmitting stimulation requests. Digital triggers sent from the stimulator can be used to synchronize acquisition systems with the stimulation clock cycle.

A stimulation pattern is a 16-bit block of data comprised of a channel ID and a waveform ID. When a request is triggered, the waveform ID is routed by the demultiplexer M1 (Figure 4) to one of 64 FIFO structures corresponding to the correct channel.

Data bus:

The digital controller communicates with the demultiplexer analog circuitry via a 16-bit data bus.

bit index	15	14	13:8	7:0
content	unused	SH control	channel	data

The demultiplexer circuitry routes the data bits to the appropriate channel using the channel bits. The SH control bit is the control signal for the sample and hold IC. After signal demultiplexing, the samples are converted to analog stimulation signals. The encoding of 64 different data streams onto the data bus is discussed in the next section.

Demultiplexing and digital to analog conversion:

The FPGA continuously outputs voltage values and their assigned channels. We only use one DAC in the system. The voltage value on the data bus gets continuously converted to an analog time-multiplexed signal. Each channel has a sample and hold IC which is only enabled when the channel value on the data bus matches the channel to which the sample and hold chip is assigned. In this way, the analog values only update for the appropriate channel.

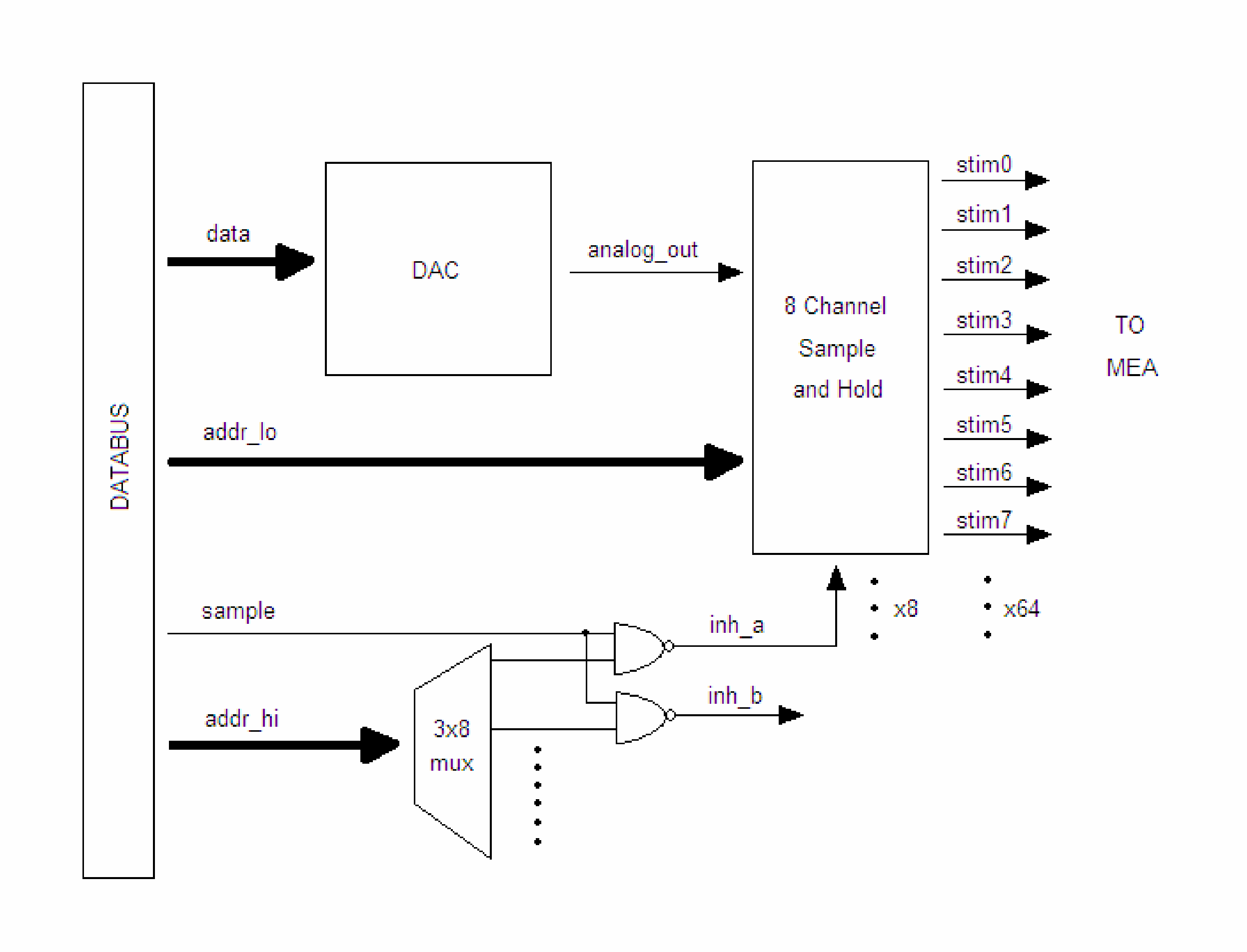

Signal descriptions:

***addr_lo* (3 bits):** Specifies to which channel within a block of eight the current stimulation signal will be directed (if a given 8-channel S&H is enabled)
***addr_hi* (3 bits):** Indicates which block (set of 8 channels) will sample the current DAC output. Specifying a block and channel chooses exactly one electrode to receive a particular stimulus.
***data* (8 bits):** Specifies the amplitude of a stimulus during a given time window.
***inh_a-inh_h* (1 bit each):** 1 bit goes to each of 8 8-channel sample-and-holds, so that up to one is enabled at a time and samples the current DAC output. These signals are the "nand" of each output bit of the 3x8 bit mux (selecting an 8-channel block) and the *sample* signal. Therefore, if the *sample* signal is deasserted, none of the 8-channel sample and holds will be sampling the current DAC output.
***sample* (1 bit):** Only when this signal is asserted *and* a particular block is selected by the current *addr_hi* signal will a particular 8-channel S&H sample the analog output of the DAC. This signal is asserted slightly after *data* is updated (specifically, the delay is equal to the propagation delay of the DAC), ensuring that the S&H is sampling a valid DAC output.
***analog_out*:** Analog output of the DAC whose amplitude is determined by the 8-bit digital input. The range of values this signal can take is determined by the potentiometers at the output of the DAC.
***stim0-stim63* (analog):** Analog signals sent to each electrode of MEA.

Digital/analog converter:

One of the useful features of this device is the ability for the user to choose the amplitude or stimulation strength of the signal sent to each electrode during each clock cycle. Each digital bit transmitted from the computer can take on one of only two possible values, a high voltage level, VH, and a low voltage level, VL. Since more flexibility in the range of possible stimulus amplitudes is obviously desirable, the DAC is used to generate a wider range of analog outputs given 8 bits of data specifying the signal amplitude. 8 bits of data allows for 256 possible values, so the DAC output is one of 256 equally spaced values between two given voltage levels. As illustrated in the above figure, only one DAC will be used to generate analog signals to all 64 electrodes. The TLC7628 DAC was chosen in this design for its low propagation delay and settling time (t_p + t_s = 180ns). It is set up in its bipolar configuration, so the analog output can take both positive and negative values. In bipolar configuration, a data input of 8'b00000000 specifies an analog output of -Vmax, an input of 8'b11111111 specifies an analog output of +Vmax, and an input of 8'b10000000 specifies an analog output of 0V.

8-channel sample and hold:

Since only one DAC chip is used for the entire system, and thus only one analog stimulus is transmitted at a time, the system needs to somehow hold the analog output at one channel as the output is being modified at other channels. Each 8-channel sample and hold chip (8Ch S&H) is capable of sampling an analog value at one of its eight outputs (although the above figure only depicts one 8Ch S&H, the system actually has eight, allowing for 64 electrodes to be stimulated). Each 8Ch S&H takes as inputs an *inhibit* signal (1 bit), three channel_select bits (*addr_lo)*, and an analog input. If *inhibit* is deasserted, the 8Ch S&H samples the DAC output and streams the signal to the output channel selected by the channel_select bits. All seven unselected channels are in "hold mode," meaning each holds the last value sampled by that channel. When *inhibit* is asserted, all 8 channels are in "hold mode." SMP18, the high speed version of the 8-channel sample and hold, was chosen in our design, again because of its relatively small propagation delay.

3-to-8 multiplexer:

Since only one DAC is used to generate analog signals for up to 64 electrodes, addressing information is needed to channel each signal appropriately. The first stage of signal channeling directs the DAC output to one of eight 8Ch S&H. The 3x8 mux takes three bits of input (specifying one of eight possible values) and outputs 8 bits (out0-out7), only one of which is asserted at a given time. For example, if the 3-bit input is 011 (corresponding to a decimal value of 3), then out3 will be asserted and out0-out2 and out4-out7 will be de-asserted. Each output 8 bit of the mux "nanded" with *sample* acts as the *inhibit* signal for one of the 8Ch S&H. This setup allows no more than one 8Ch S&H to be enabled at once.

Signal conditioning:

Finally, sample-and-hold channels are fed through a final-stage op-amp for signal conditioning on each channel (OP495, Analog Electronics). A few additional op-amps, or/nor gates, and 146 voltage overrides are scattered throughout the circuit for further optimization.

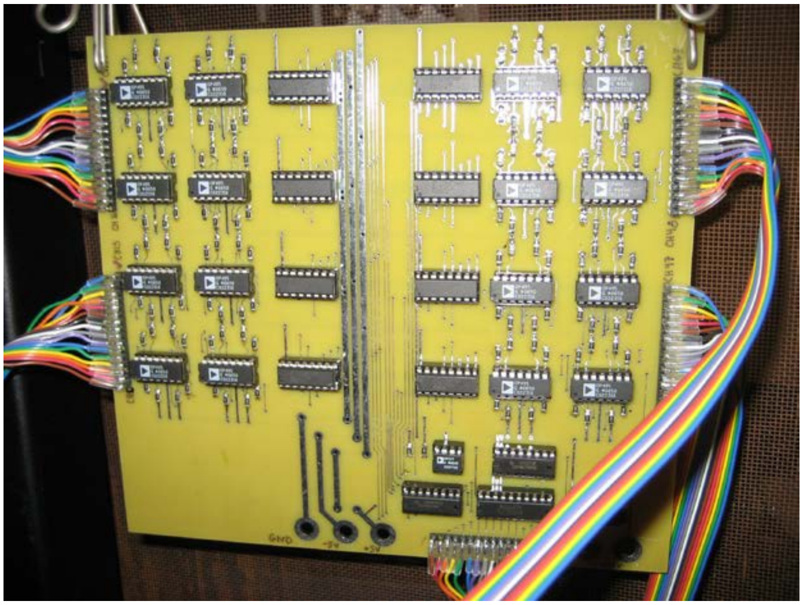

Resultant analog stimulation

The amplitude of a stimulus at a given time is determined both by the eight data bits from the data bus as well as by the DAC's external potentiometers which set the DAC's gain and will be delivered and instantaneously stored by the sample and hold addressed by the routing bits. The system will cycle through all sample and holds, loading them up with their next analog voltage, and once all channels have been updated it will cycle through the next time point of each. In this way a steady stream of temporally precise analog voltage levels are dispatched across a large number of channels. In principle this design can also be readily scaled, doubling channels with each new address bit added to the routing logic.

Connecting from the analog circuitry to the multi-electrode array:

The output of the 64 channels arising from the sample-and-holds was wired into a unified SCSI connector via a National Instruments SCB-68 connector box. This in turn connected to a 68-pin SCSI cable that came with a MultiChannel Systems MEA60 connector platform, and which connects to its built-in connector port. A planar multi-electrode array chip from MultiChannel Systems was then loaded into the connector platform, thus enabling end-to-end communication channels from a software environment into an electrode-rich stimulation area.

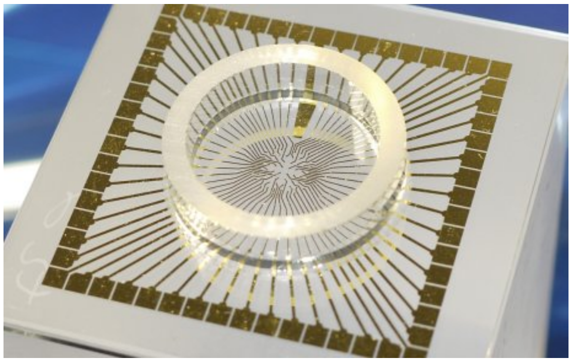

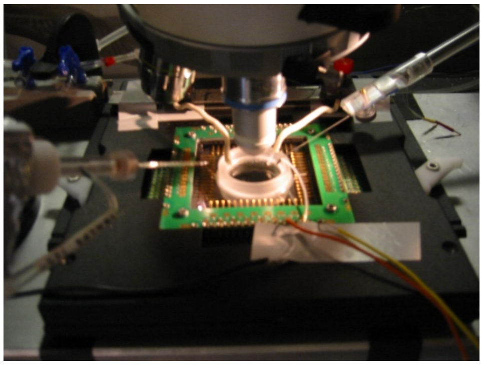

Supplement: Supplementary file 7 [file Data_Sheet_7.PDF]
